# *Arabidopsis* downy mildew effector HaRxL106 suppresses plant immunity by binding to RADICAL‐INDUCED CELL DEATH1

**DOI:** 10.1111/nph.15277

**Published:** 2018-08-29

**Authors:** Lennart Wirthmueller, Shuta Asai, Ghanasyam Rallapalli, Jan Sklenar, Georgina Fabro, Dae Sung Kim, Ruth Lintermann, Pinja Jaspers, Michael Wrzaczek, Jaakko Kangasjärvi, Daniel MacLean, Frank L. H. Menke, Mark J. Banfield, Jonathan D. G. Jones

**Affiliations:** ^1^ The Sainsbury Laboratory Norwich Research Park Norwich NR4 7UH UK; ^2^ Dahlem Centre of Plant Sciences Department of Plant Physiology and Biochemistry Freie Universität Berlin Königin‐Luise‐Straße 12–16 14195 Berlin Germany; ^3^ Division of Plant Biology Department of Biosciences University of Helsinki FIN‐00014 Helsinki Finland; ^4^ Department of Biological Chemistry John Innes Centre Norwich Research Park Norwich NR4 7UH UK

**Keywords:** *Arabidopsis thaliana*, *Hyaloperonospora arabidopsidis*, oomycete pathogen, pathogen effector, plant innate immunity, RADICAL‐INDUCED CELL DEATH1, salicylic acid (SA)

## Abstract

- The oomycete pathogen *Hyaloperonospora arabidopsidis* (*Hpa*) causes downy mildew disease on *Arabidopsis*. To colonize its host, *Hpa* translocates effector proteins that suppress plant immunity into infected host cells. Here, we investigate the relevance of the interaction between one of these effectors, HaRxL106, and *Arabidopsis* RADICAL‐INDUCED CELL DEATH1 (RCD1).
- We use pathogen infection assays as well as molecular and biochemical analyses to test the hypothesis that HaRxL106 manipulates RCD1 to attenuate transcriptional activation of defense genes.
- We report that HaRxL106 suppresses transcriptional activation of salicylic acid (SA)‐induced defense genes and alters plant growth responses to light. HaRxL106‐mediated suppression of immunity is abolished in *RCD1* loss‐of‐function mutants. We report that RCD1‐type proteins are phosphorylated, and we identified Mut9‐like kinases (MLKs), which function as phosphoregulatory nodes at the level of photoreceptors, as RCD1‐interacting proteins. An *mlk1,3,4* triple mutant exhibits stronger SA‐induced defense marker gene expression compared with wild‐type plants, suggesting that MLKs also affect transcriptional regulation of SA signaling.
- Based on the combined evidence, we hypothesize that nuclear RCD1/MLK complexes act as signaling nodes that integrate information from environmental cues and pathogen sensors, and that the *Arabidopsis* downy mildew pathogen targets RCD1 to prevent activation of plant immunity.

## Introduction

Plants rely on their innate immune system to distinguish beneficial microbes from harmful pathogens or commensal bacteria. While plant innate immunity fends off the majority of attempted infections, specialized pathogens can subvert host defenses with effector proteins that are translocated into host cells. Many pathogen effectors interfere with cellular processes that are essential for innate immunity, such as formation of cell wall appositions, secretion of antimicrobial compounds, production of reactive oxygen species (ROS), or transcriptional activation of defense genes (DebRoy *et al*., 2004; Nomura *et al*., 2006; Bozkurt *et al*., 2011; Anderson *et al*., 2012; Gangadharan *et al*., 2013; Asai *et al*., 2014). Bacterial pathogens have evolved specialized secretion systems to deliver effectors into host cells (Deng *et al*., 2017). Likewise, the fungal rice blast pathogen *Magnaporthe oryzae* employs a specialized secretion pathway to deliver host‐cell‐targeted effectors into a host‐derived compartment named the biotrophic interfacial complex (Khang *et al*., 2010; Giraldo *et al*., 2013). How other filamentous plant pathogens, such as oomycetes, translocate effectors into plant cells remains poorly understood (Petre & Kamoun, 2014; Lo Presti & Kahmann, 2017). Like fungal pathogens, oomycetes elaborate haustoria, bulbous feeding structures that induce the formation of a specialized extra‐haustorial membrane, within the plasma membrane of infected host cells (Lo Presti & Kahmann, 2017). Notably, haustoria are also sites of targeted effector secretion (Whisson *et al*., 2007; Gilroy *et al*., 2011; Liu *et al*., 2014b; Wang *et al*., 2017). Most host‐cell‐targeted oomycete effectors carry a combination of a signal peptide and a conserved amino acid motif RXLR (where X represents any amino acid). The RXLR motif is required for effector translocation into the host cell, and there is evidence that it functions as an internal sorting signal (Grouffaud *et al*., 2008; Wawra *et al*., 2017). The effector protein domains downstream of signal peptide and RXLR motif are diverse and constitute the part of the effector that manipulates cellular processes in the host cell (Franceschetti *et al*., 2017).

Plants respond to infection by biotrophic pathogens with elevated biosynthesis of the defense hormone salicylic acid (SA), and elevated SA levels lead to thioredoxin‐catalyzed reduction of disulfide‐linked oligomeric complexes of the NONEXPRESSOR OF PATHOGENESIS‐RELATED GENE 1 (NPR1) protein (Mou *et al*., 2003; Tada *et al*., 2008). Monomeric NPR1 translocates to the nucleus, where it functions as a transcriptional co‐activator and is indispensable for SA responsiveness of many SA‐induced genes (Wang *et al*., 2006). Some biotrophic plant pathogen effectors actively suppress SA accumulation and/or SA signaling. The maize smut fungus *Ustilago maydis* produces a host‐cell‐targeted chorismate dismutase that may suppress SA‐mediated immunity by diverting the SA‐precursor chorismate into the phenylpropanoid pathway (Djamei *et al*., 2011). The oomycete pathogen *Phytophthora sojae* and the fungal pathogen *Verticillium dahliae* attenuate SA signal transduction by delivery of isochorimatases into host cells (Liu *et al*., 2014b). The host‐targeted effector Pi04314 from the late blight pathogen *Phytophthora infestans* targets several nuclear‐localized phosphatases and attenuates the transcriptional response to SA and methyl jasmonate (MeJA) (Boevink *et al*., 2016). The *Arabidopsis* downy mildew pathogen *Hyaloperonospora arabidopsidis* (*Hpa*) also suppresses transcriptional upregulation of the SA marker gene *PATHOGENESIS‐RELATED GENE 1* (*PR1*) in infected host cells (Caillaud *et al*., 2013). At least two *Hpa* effector proteins interfere with SA signaling when expressed as transgenes in *Arabidopsis*. Effector HaRxL44 appears to attenuate SA signal transduction by targeting the MEDIATOR subunit Med19 for proteasomal degradation (Caillaud *et al*., 2013), while effector HaRxL62 interferes with SA signaling by an unknown mechanism (Asai *et al*., 2014).

Light perception and signaling also influence the transcriptional response to SA (Genoud *et al*., 2002; de Wit *et al*., 2013; Gangappa *et al*., 2016). Simulated shade conditions, for example, suppress transcript changes induced by exogenous application of SA or MeJA, thereby attenuating plant defense toward biotrophic and necrotrophic pathogens (Izaguirre *et al*., 2006; Cerrudo *et al*., 2012; de Wit *et al*., 2013). This is remarkable given that SA‐ and jasmonic acid (JA)‐responsive gene networks are antagonistically regulated in response to infection by pathogens with either a biotrophic or necrotrophic mode of infection (Pieterse *et al*., 2012; Caarls *et al*., 2015).

*Arabidopsis* RADICAL‐INDUCED CELL DEATH1 (RCD1) has been proposed to act as a positive regulator of SA signaling. Loss of *RCD1* function does not alter SA levels, but transcript levels of many NPR1 target genes are lower in *rcd1* mutants compared with wild‐type plants (Ahlfors *et al*., 2004; Brosché *et al*., 2014). *RCD1* was initially identified in a screen for ozone‐sensitive *Arabidopsis* mutants. The *rcd1‐1* mutant is impaired in restricting programmed cell death under sublethal ozone concentrations (Overmyer *et al*., 2000). In addition, *rcd1* mutants show pleiotropic phenotypes that include a smaller rosette size and altered leaf shape, as well as partial loss of apical dominance and an altered root system architecture (Ahlfors *et al*., 2004; Teotia & Lamb, 2009). Loss of *RCD1* function enhances sensitivity to apoplastic ROS and salt stress but increases tolerance to chloroplastic ROS, and this correlates with altered transcription of genes that are responsive to ROS, abscisic acid, JA, ethylene, and SA (Ahlfors *et al*., 2004; Overmyer *et al*., 2005; Katiyar‐Agarwal *et al*., 2006; Brosché *et al*., 2014).

RCD1 is the founding member of a plant‐specific protein family characterized by a central domain with sequence similarity to the catalytic domain of Poly‐(ADP‐ribose)‐polymerases (PARPs) (Lamb *et al*., 2012). In contrast to canonical PARPs that covalently modify target proteins by ADP‐ribosylation, *Arabidopsis* RCD1 does not show PARP activity *in vitro* when expressed as a GST fusion (Jaspers *et al*., 2010). However, an RCD1 homologue from wheat shows PARP activity when expressed in *Escherichia coli*, suggesting that some RCD1‐type proteins may be enzymatically active (Liu *et al*., 2014a). In addition to the central PARP domain, RCD1 and its paralogue SIMILAR TO RCD ONE1 (SRO1) have an N‐terminal WWE domain and a C‐terminal RST domain. RCD1 and sequence‐related proteins localize to the plant cell nucleus and bind several transcription factors via their RST domains, possibly explaining why loss of *RCD1* function affects plant development and several stress signaling pathways (Katiyar‐Agarwal *et al*., 2006; Jaspers *et al*., 2009; You *et al*., 2014). Accordingly, RCD1 might influence SA signal transduction by interacting with transcription factors that control expression of defense genes.

RCD1 interacts with the *Hpa* effector HaRxL106 in a yeast‐two‐hybrid (Y2H) assay, and this effector renders *Arabidopsis* more susceptible to biotrophic pathogens when expressed as a transgene (Fabro *et al*., 2011; Mukhtar *et al*., 2011). In plant cells, HaRxL106 is actively transported into the nucleus, indicative of a nuclear virulence‐promoting activity of the effector (Wirthmueller *et al*., 2015). Here, we report that HaRxL106, when expressed as a transgene, affects both SA signaling and light‐regulated developmental processes. We identify RCD1 as a likely virulence target of HaRxL106 and report that RCD1 interacts with Mut9‐like kinases (MLKs) that, in addition to their previously characterized function in light signal transduction, also influence the transcriptional response to SA.

## Materials and Methods

### Plants and growth conditions

For hypocotyl growth assays, *Arabidopsis* seeds were sown on Murashige‐Skoog medium (Duchefa #M0255) containing 0.1 g l^−1^ myoinositol and 8 g l^−1^ Bactoagar, stratified for 48 h at 4°C in the dark. Germination was induced by a 6 h white light stimulus. The plates were placed in long‐day (12 h : 12 h, light : dark) conditions at 21°C and a fluence rate of 12 μmol m^−2^ s^−1^ white light. Hypocotyl length was determined on day 5 using ImageJ software. Growth conditions for *Nicotiana benthamiana* and all other *Arabidopsis* experiments were as in Segonzac *et al*. (2011) and Fabro *et al*. (2011). The *rcd1‐1* and *rcd1‐3* mutants have been described (Palma *et al*., 2005; Gao *et al*., 2009; Jaspers *et al*., 2009; Yang *et al*., 2017). The *mlk1,2,3* and *mlk1,3,4* triple mutants have been described (Huang *et al*., 2016).

### Generation of transgenic *Arabidopsis* lines

Transgenic *Arabidopsis* plants expressing yellow fluorescent protein (YFP)‐tagged HaRxL106 have been described (Wirthmueller *et al*., 2015). To generate transgenic lines expressing 3xHA‐StrepII (HS):HaRxL106, RFP:HaRxL106, RFP:NLS:HaRxL106ΔC, and RFP:HaRxL106‐Cterm58 (all *Hpa* Emoy2) we used the following previously described pENTR plasmids: pENTR4‐HaRxL106, M followed by HaRxL106 amino acids I^25^–S^285^ (Fabro *et al*., 2011), pENTR/D‐TOPO‐SV40NLS:HaRxL106ΔC, sequence APKKKRKV followed by HaRxL106 amino acids I^25^–G^229^, and pENTR/D‐TOPO‐HaRxL106‐Cterm58, HaRxL106 amino acids G^228^–S^285^ (Wirthmueller *et al*., 2015). Plasmids pXCSG‐HS and pH7WGR2 were recombined with the aforementioned pENTR plasmids using Gateway^®^ LR clonase II to generate HS‐ and red fluorescent protein (RFP)‐tagged versions of the HaRxL106 constructs respectively. Transgenic HS‐tagged HaRxL106 lines were generated by transforming Col‐0 or *35S*_*Pro*_ *:NPR1:GFP* plants with *Agrobacterium tumefaciens* GV3101::pMP90RK carrying pXCSG‐*HS:HaRxL106* constructs using floral dip (Logemann *et al*., 2006). RFP‐tagged HaRxL106 lines were generated by transforming Col‐0 with *A. tumefaciens* GV3101::pMP90 carrying the corresponding pH7WGR2 plasmids. Col‐0 lines expressing RCD1 amino acids 1–265 as C‐terminal fusion to green fluorescent protein (GFP) were generated by recombining a corresponding pENTR clone with pK7WGF2 (Karimi *et al*., 2002); the construct was transformed into Col‐0 plants as earlier. Site‐directed mutants of RCD1 were generated using the QuikChange method (Agilent, Santa Clara, CA, USA). pENTR/D‐TOPO plasmids carrying the mutated RCD1 variants in fusion with 2499 bp of the *RCD1* promoter sequence were recombined with pGWB13 (Nakagawa *et al*., 2007) to generate a translational fusion with a triple HA‐tag at the C‐terminus of the protein. The constructs were transformed into the *rcd1‐1* mutant.

### *Hpa* infection and quantification

**Figure 1:**
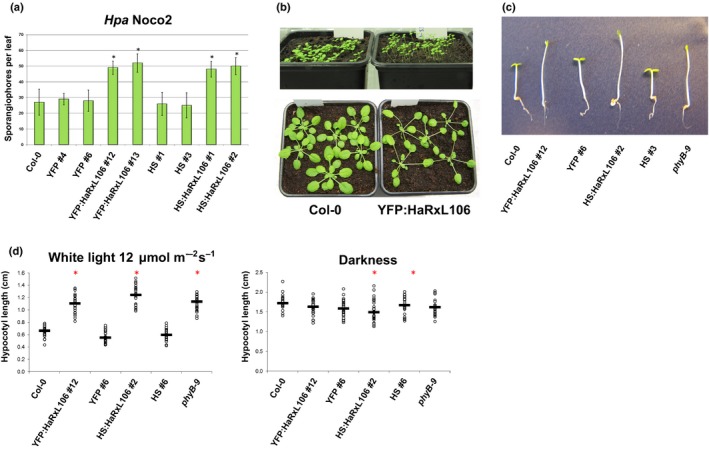
(a) Sporulation of the virulent *Hyaloperonospora arabidopsidis* isolate Noco2 on 6‐wk‐old *Arabidopsis thaliana* plants of the indicated genotypes quantified by the number of conidiophores per leaf. The results shown are representative of two independent biological experiments, *n *=* *20, error bars show plus/minus SEM, and asterisks indicate differences from Col‐0 (one‐way ANOVA; Tukey–Kramer post‐hoc test, *P *<* *0.05). See Supporting Information Table S1 for source data and statistics. (b) Constitutive expression of HaRxL106 induces phenotypes that are reminiscent of the shade avoidance syndrome in *Arabidopsis thaliana*. The top panel shows 10‐d‐old seedlings of Col‐0 and a representative *35S*_*Pro*_ *:YFP:HaRxL106* line. Bottom panel shows 4‐wk‐old plants from both genotypes grown under short day condition and a fluence rate of *c*. 120 μmol m^−2^ s^−1^. (c) Five‐day‐old seedlings of the indicated genotypes germinated under a lower fluence rate of *c*. 12 μmol m^−2^ s^−1^. (d) Quantification of seedling hypocotyl length of the indicated genotypes grown as in (c) or in darkness. The results shown are representative of three independent biological experiments, *n *=* *30, horizontal bars denote median, and asterisks indicate mean values different from Col‐0 (one‐way ANOVA; Tukey–Kramer post‐hoc test, *P *<* *0.05). See Table S2 for source data and statistics.

Mutants and transgenic lines were tested for altered susceptibility to *Hpa* Noco2 either in adult leaves of 6‐wk‐old plants (Fig. 1a) or in cotyledons of 10‐d‐old seedlings grown on soil. For both types of experiments, plants were sprayed with a suspension of 1 × 10^5^ spores ml^−1^. The plants were placed in high (> 90%) humidity under a plastic dome. Sporulation on seedlings was scored at 5 d post infection, and sporulation on adult plants was quantified 7–8 d post infection. For the adult leaf assay, 20 leaves per genotype were stained with trypan blue. Following destaining with chloral hydrate solution, conidiophores on 20 leaf areas of 1 cm^2^ were counted using a light microscope. For the seedling assay, 35–40 seedlings per genotype were incubated in a 0.02% (w/v) Uvitex 2B (Polysciences, Hirschberg an der Bergstrasse, Germany) solution, then destained in water for 2 min, mounted on a Styrofoam rack, and imaged through a Leica UV filter (Leica #10447415) using a Leica M165 FC fluorescent stereomicroscope connected to an EL6000 laser source. Only conidiophores on the upper side of the cotyledons were counted.

### SA treatment

For SA treatment, 4‐wk‐old *Arabidopsis* plants were sprayed with a solution containing 0.1 mM SA and 0.01% Silwet L‐77 1 h after dawn (09:00 h). Rosette leaves were harvested 8 h later.

## Results

### HaRxL106‐expressing *Arabidopsis* plants exhibit attenuated light and defense signaling

To characterize HaRxL106‐interacting proteins from *Arabidopsis* we generated transgenic lines expressing HaRxL106 with an N‐terminal YFP or 3xHA‐StrepII (HS) epitope tag under control of the *35S* promoter. As previously reported for transgenic plants expressing untagged HaRxL106 (Fabro *et al*., 2011), these lines are hyper‐susceptible to infection by the compatible *Hpa* isolate Noco2 (Fig. 1a; Supporting Information Table S1). Notably, lines expressing HaRxL106 show a phenotype reminiscent of plants grown under shade; specifically, longer hypocotyls and elongated petioles (Fig. 1b). Differences in hypocotyl length between wild‐type plants and transgenic lines were more pronounced when we grew seedlings under a lower fluence rate of white light (12 μmol m^−2^ s^−1^) (Fig. 1c,d; Table S2). Under these conditions, HaRxL106‐expressing seedlings were indistinguishable from the *phyB‐9* mutant that shows constitutive shade avoidance (Reed *et al*., 1993). Lines expressing control constructs YFP and HS did not differ from wild‐type plants in hypocotyl length (Fig. 1c,d). By contrast, differences in hypocotyl length between HaRxL106‐expressing transgenic lines and wild‐type plants were much smaller when we grew seedlings in darkness (Fig. 1d; Table S2). This suggests that, in addition to suppression of plant immunity, HaRxL106 also affects signal transduction between photoreceptors and light‐regulated elongation growth.

### Effector HaRxL106 suppresses SA signal transduction but not SA levels

**Figure 2:**
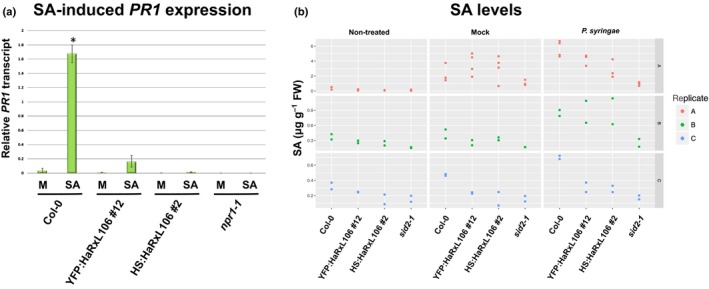
(a) *Hyaloperonospora arabidopsidis* effector HaRxL106 suppresses salicylic acid (SA)‐induced *PATHOGENESIS‐RELATED GENE 1* (*PR1*) expression in *Arabidopsis thaliana*. Four‐week‐old plants of the indicated genotypes were sprayed with 0.1 mM SA or a mock (M) solution and *PR1* expression levels were analyzed by quantitative real‐time PCR 8 h later. *PR1* expression levels were normalized by *ELONGATION FACTOR 1 ALPHA* (*EF1α*) expression. The plot shows the mean of *PR1/EF1α* expression from three independent biological experiments. Error bars show plus/minus SEM; asterisk indicates mean value different from Col‐0 mock treatment (one‐way ANOVA; Tukey–Kramer post‐hoc test, *P *<* *0.05). See Supporting Information Table  S3 for source data and statistics. (b) Quantification of free SA levels in the indicated genotypes under nontreated conditions and 24 h after infiltration with 10^8^ CFU ml^−1^ of *Pseudomonas syringae* pv. tomato DC3000 or a 10 mM magnesium chloride mock solution. Red, green, and blue represent data from three independent biological experiments. Dots of the same color represent technical replicates. See Table S4 for source data and statistics.

As *phyB* mutants show an attenuated transcriptional response to SA (Genoud *et al*., 2002; de Wit *et al*., 2013), and given that suppression of SA signal transduction would be a conceivable virulence mechanism for an effector from a biotrophic pathogen, we tested SA‐induced upregulation of the defense marker gene *PR1* in Col‐0 plants and two transgenic lines, one expressing YFP:HaRxL106 and the other HS:HaRxL106. SA induced *PR1* mRNA levels in Col‐0 plants but not in the *npr1‐1* mutant (Cao *et al*., 1994) (Fig. 2a; Table S3). By contrast, *PR1* expression levels in SA‐treated HaRxL106 transgenic lines were comparable to those in mock‐treated Col‐0 plants, suggesting that HaRxL106 affects either endogenous SA levels or SA signal transduction (Fig. 2a). Quantification of unconjugated SA levels in leaves 24 h after infiltration of *Pseudomonas syringae* pv. tomato (*Pst*) DC3000 revealed that SA concentrations in HaRxL106‐expressing lines were comparable to Col‐0 (one‐way ANOVA; *P *=* *0.648) (Fig. 2b; Table S4). These results suggest that HaRxL106 does not substantially alter SA levels but nevertheless strongly attenuates SA‐induced transcriptional regulation of the SA marker gene *PR1*.

### Effector HaRxL106 attenuates NPR1‐dependent defense activation

**Figure 3:**
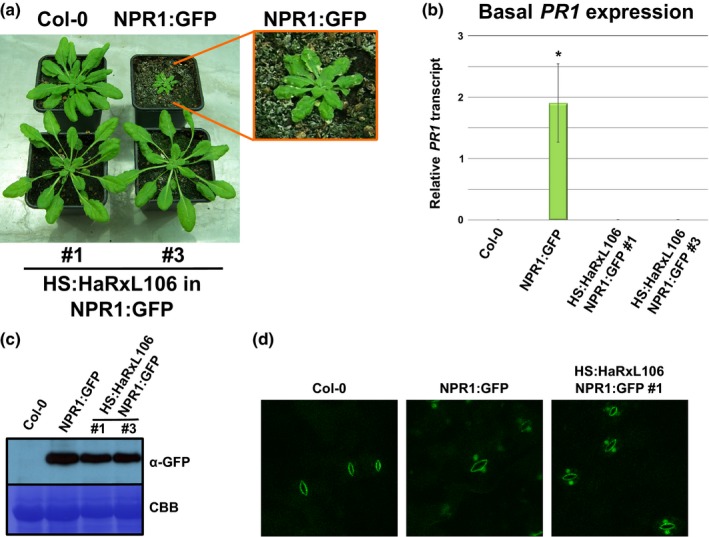
*Hyaloperonospora arabidopsidis* effector HaRxL106 suppresses constitutive defense signaling induced by NPR1:GFP overexpression in *Arabidopsis thaliana* under short day conditions. (a) Morphology of 5‐wk‐old Col‐0 and *35S*_*Pro*_ *:NPR1:GFP* plants (top row) and two independent double transgenic *35S*_*Pro*_ *:NPR1:GFP* lines co‐expressing *35S*_*Pro*_ *:HS:HaRxL106* (bottom row). The inset shows spontaneous lesions forming in *35S*_*Pro*_ *:NPR1:GFP* plants. (b) Basal *PATHOGENESIS‐RELATED GENE 1* (*PR1*) expression in the lines shown in (a) as determined by quantitative real‐time PCR. *PR1* expression levels were normalized by *ELONGATION FACTOR 1 ALPHA* (*EF1α*) expression. The plot shows the mean of *PR1/EF1α* expression from three independent biological experiments. Error bars show ± SEM, asterisk indicates mean value different from Col‐0 (one‐way ANOVA; Tukey‐Kramer post‐hoc test, *P *<* *0.05). See Table S5 for source data and statistics. (c) Western blot showing accumulation of NPR1:GFP protein in the lines shown in (a). The Western blot is representative of three independent biological experiments. (d) Representative (*n *>* *10) confocal microscopy images showing nuclear accumulation of NPR1:GFP protein in short day conditions in *35S*_*Pro*_ *:NPR1:GFP* plants and plants co‐expressing *35S*_*Pro*_ *:HS:HaRxL106*. The signal in Col‐0 is auto‐fluorescence from stomata.

HaRxL106 is actively transported into nuclei of plant cells (Wirthmueller *et al*., 2015). Given that NPR1 is an important nuclear signal integrator of the SA pathway, we tested whether HaRxL106 affects NPR1 localization or protein levels. When plants expressing *35S*_*Pro*_ *:NPR1:GFP* in an *npr1‐1* mutant background (Kinkema *et al*., 2000) are grown under short day conditions the plants show signs of constitutive defense activation, including severe stunting, development of micro lesions, and elevated *PR1* expression (Fig. 3a,b; Table S5; Love *et al*., 2012). We transformed the *35S*_*Pro*_ *:NPR1:GFP* line with the *35S*_*Pro*_ *:HS:HaRxL106* construct and found that expression of HaRxL106 completely suppressed the stunting of the *35S*_*Pro*_ *:NPR1:GFP* line in 12 out of 14 independent transgenic lines (Fig. 3a). HaRxL106 also reverted the constitutive *PR1* expression of the *35S*_*Pro*_ *:NPR1:GFP* line (Fig. 3b). This suppression was not due to lower NPR1:GFP protein levels, as shown by the western blot in Fig. 3c. Consistent with constitutively activated defense, we observed nuclear localization of NPR1:GFP in guard cells of plants grown under short day condition even without exogenous SA application (Fig. 3d). NPR1:GFP also localized to nuclei in double transgenic lines co‐expressing HS:HaRxL106 (Fig. 3d). Taken together, these results show that HaRxL106 does not attenuate SA signal transduction by altering protein levels or localization of NPR1. As HaRxL106 suppresses constitutive *PR1* gene expression induced by the *35S*_*Pro*_ *:NPR1:GFP* transgene, the effector must either act downstream of nuclear NPR1 signaling or disrupt the nuclear transactivator function of NPR1 itself.

### HaRxL106‐overexpressing lines show a partial transcription profile overlap with the *radical‐induced cell death1‐1* mutant

**Figure 4:**
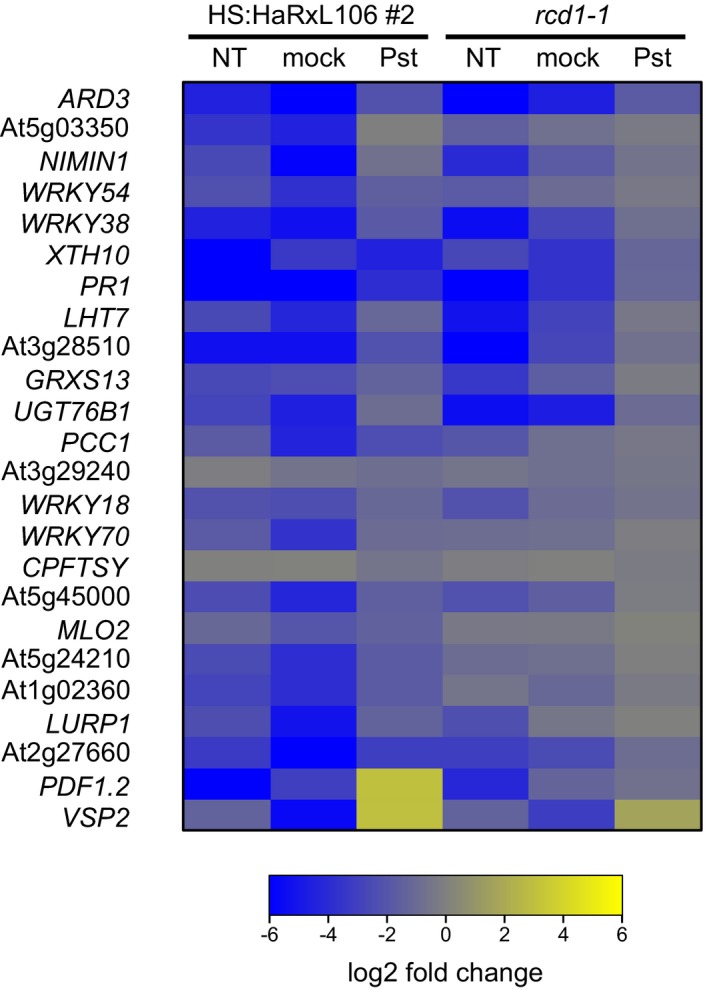
Expression levels of salicylic acid (SA)‐responsive defense genes and the jasmonic acid (JA) marker genes *PDF1.2* and *VSP2* in *Arabidopsis thaliana* HS:HaRxL106 line #2 and the *rcd1‐1* mutant. NT, nontreated plants; mock, 24 h after infiltration of 10 mM magnesium chloride; Pst, 24 h after infiltration with 5 × 10^5^ CFU ml^−1^ *Pseudomonas syringae* pv. tomato DC3000. Blue and yellow colors denote the level of down‐ and upregulation respectively in comparison with expression levels in Col‐0. See Supporting Information Table S6 for source data and false discovery rates.

Similar to HaRxL106‐expressing lines, *rcd1* mutants show lower expression levels of *PR1*, as well as other SA marker genes (Brosché *et al*., 2014). As the two proteins interact in Y2H, RCD1 could be a virulence target of HaRxL106. We performed a transcriptome profiling experiment to characterize defense gene expression of a representative HS:HaRxL106‐expressing line and the *rcd1‐1* mutant in more detail (Methods S1). We profiled complementary DNA from nontreated plants as well as complementary DNA prepared from leaves that were infiltrated with *Pst* DC3000 or a magnesium chloride mock control 24 h earlier. As shown in Fig. S1, the *rcd1‐1* mutant showed a partial transcriptional overlap with the HaRxL106‐expressing line, particularly for repressed genes under nontreated conditions. A functional classification of this gene set using Gene Ontology terms revealed an overrepresentation of SA‐responsive defense genes (Table S6). Fig. 4 shows the expression levels of 22 SA/NPR1‐regulated genes (Wang *et al*., 2006) and the JA marker genes *PDF1.2* and *VSP2* in the HaRxL106‐expressing line and *rcd1‐1*. In nontreated and mock‐treated plants, SA marker genes were repressed compared to Col‐0. Expression levels of the two JA marker genes were either repressed or not different from wild‐type, and this pattern is reminiscent of shade‐grown plants (de Wit *et al*., 2013). At 24 h after infection with *Pst* DC3000, expression levels of SA marker genes in *rcd1‐1* were similar to Col‐0, while 16 out of 22 SA marker genes remained repressed in the HaRxL106‐expressing line (Fig. 4; Table S6). Therefore, the enhanced susceptibility mediated by ectopic expression of HaRxL106 is likely due to its repressive effect on transcription of SA‐responsive defense genes. Loss of *RCD1* function results in a comparably low expression level of SA marker genes before pathogen challenge. However, defense genes in *rcd1‐1* are still transcriptionally induced upon bacterial infection, resulting in a defense transcriptome that is more similar to wild‐type plants 24 h after infection (Fig. 4; Table S6). These results suggest that, if RCD1 is a virulence target of HaRxL106, the manipulative effect of HaRxL106 is not mimicked by a complete loss of *RCD1* function.

### HaRxL106 interacts with RCD1 and SRO1 proteins and RCD1 quantitatively contributes to SA signal transduction

**Figure 5:**
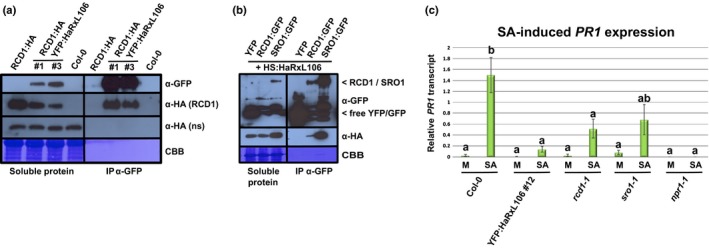
*Hyaloperonospora arabidopsidis* effector HaRxL106 interacts with *Arabidopsis thaliana* RADICAL‐INDUCED CELL DEATH1 (RCD1) and SIMILAR TO RCD ONE1 (SRO1) in plant cells and RCD1 contributes to salicylic acid (SA)‐induced *PATHOGENESIS‐RELATED GENE 1* (*PR1*) expression. (a) Functional RCD1:HA protein co‐immunoprecipitates with YFP:HaRxL106 in *Arabidopsis*. YFP:HaRxL106 was immunoprecipitated from double transgenic lines expressing RCD1:HA, proteins were resolved by sodium dodecyl sulfate polyacrylamide gel electrophoresis (SDS‐PAGE), transferred onto polyvinylidene fluoride (PVDF) membrane and probed with the indicated antibodies. ns, nonspecific band detected by the α‐HA antibody. CBB, Coomassie brilliant blue stain. This result is representative of three independent biological experiments. (b) HS:HaRxL106 co‐immunoprecipitates with GFP‐tagged variants of RCD1 and SRO1 following transient expression in *Nicotiana benthamiana*. GFP‐tagged proteins, or YFP as a control, were immunoprecipitated, proteins were resolved by SDS‐PAGE, transferred onto PVDF membrane, and probed with the indicated antibodies. Co‐immunoprecipitation of HaRxL106 with RCD1 and SRO1 is based on three and two independent biological experiments respectively. (c) SA‐induced *PR1* gene expression in Col‐0, YFP:HaRxL106 line #12, *rcd1‐1*,* sro1‐1*, and *npr1‐1* mutants. Four‐week‐old plants were sprayed with 0.1 mM SA or a mock (M) solution and *PR1* expression levels were analyzed by quantitative real‐time PCR 8 h later. *PR1* expression levels were normalized by *ELONGATION FACTOR 1 ALPHA* (*EF1α*) expression. The plot shows the mean of *PR1/EF1α* expression from five independent biological experiments. Error bars show plus/minus SEM; letters indicate differences between genotypes/treatments (one‐way ANOVA; Tukey–Kramer post‐hoc test, *P *<* *0.05). See Supporting Information Table S7 for source data and statistics.

To test for interaction between HaRxL106 and RCD1 in *Arabidopsis*, we transformed a transgenic line in which the *rcd1‐3* mutation is complemented by expression of an *RCD1*_*Pro*_ *:RCD1:HA* construct (Jaspers *et al*., 2009), with YFP:HaRxL106 and selected double transgenic lines. When we immunoprecipitated YFP:HaRxL106 from these plants, RCD1:HA co‐purified with HaRxL106, whilst a cross‐reacting band detected by the α‐HA antibody did not (Fig. 5a). Therefore, YFP:HaRxL106 interacts with functional epitope‐tagged RCD1:HA protein in *Arabidopsis*.

*RCD1* and its paralogue *SRO1* show unequal genetic redundancy with respect to plant development and responses to abiotic stress with *RCD1* making a stronger contribution (Jaspers *et al*., 2009; Teotia & Lamb, 2009). Transiently expressed, GFP‐tagged versions of RCD1 and SRO1 co‐immunoprecipitated HS:HaRxL106, whereas a YFP control did not (Fig. 5b). This suggests that HaRxL106 interacts with both RCD1 and SRO1 in plant cells. The partial redundancy between *RCD1* and *SRO1* prompted us to test whether *SRO1* also contributes to transcriptional regulation of NPR1 target genes. Overall, the *rcd1‐1* and *sro1‐1* mutations had a weaker effect on SA‐induced *PR1* expression than the YFP:HaRxL106 transgene (Fig. 5c). *PR1* levels in *rcd1‐1* but not in *sro1‐1* showed a significant reduction compared with those in Col‐0 (one‐way ANOVA, Tukey–Kramer post hoc test; *P* < 0.05) (Fig. 5c; Table S7). Therefore, *RCD1* quantitatively contributes to SA‐induced *PR1* expression. Owing to the requirement of either *RCD1* or *SRO1* for normal embryogenesis (Teotia & Lamb, 2009), we were not able to test an *rcd1 sro1* double mutant for SA‐induced *PR1* expression.

### The C‐terminal 58 amino acids of HaRxL106 are required for RCD1 binding and attenuation of light and defense signaling

**Figure 6:**
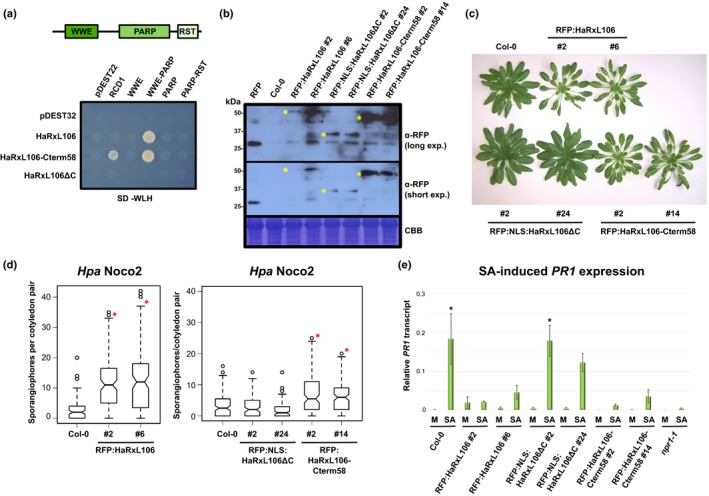
The C‐terminal 58 amino acids of *Hyaloperonospora arabidopsidis* (*Hpa*) effector HaRxL106 are required for binding to *Arabidopsis thaliana* RADICAL‐INDUCED CELL DEATH1 (RCD1) and affect light‐regulated plant growth and defense signaling. (a) Protein–protein interactions between RCD1, HaRxL106, and the indicated deletion constructs in a yeast‐two‐hybrid assay. pDEST32 and pDEST22 are empty bait and prey vectors respectively. HaRxL106ΔC is a deletion construct lacking HaRxL106's 56 C‐terminal amino acids. HaRxL106‐Cterm58 is an N‐terminal deletion construct consisting only of the 58 C‐terminal amino acids. (b) Western blot showing protein levels of RFP‐tagged HaRxL106, NLS:HaRxL106ΔC, and HaRxL106‐Cterm58 in selected stable transgenic *Arabidopsis* lines. Asterisks indicate the expected migration based on the molecular weight of the fusion proteins. (c) Visual phenotype of transgenic *Arabidopsis* lines expressing RFP‐tagged HaRxL106, HaRxL106ΔC, or HaRxL106‐Cterm58. (d) Resistance to the virulent *Hpa* isolate Noco2 in 10‐d‐old seedlings of Col‐0 and transgenics expressing RFP‐tagged HaRxL106, HaRxL106ΔC, or HaRxL106‐Cterm58. The plots show the number of conidiophores per cotyledon pair. Data from three independent biological experiments were pooled, horizontal bars show median, vertical box height represents interquartile range (IQR), and whisker range is 1.5× IQR. Circles represent data points beyond 1.5× IQR. Asterisks indicate mean values different from Col‐0 (one‐way ANOVA; Tukey–Kramer post‐hoc test, *P *<* *0.05). See Supporting Information Table S8 for source data and statistics. (e) Salicylic acid (SA)‐induced *PATHOGENESIS‐RELATED GENE 1* (*PR1*) gene expression in Col‐0, and transgenics expressing RFP‐tagged HaRxL106, NLS:HaRxL106ΔC or HaRxL106‐Cterm58. Four‐wk‐old plants were sprayed with 0.1 mM SA or a mock (M) solution and *PR1* expression levels were analyzed by qRT‐PCR 8 h later. *PR1* expression levels were normalized by *ELONGATION FACTOR 1 ALPHA* (*EF1α*) expression. The plot shows the mean of *PR1/EF1α* expression from three independent biological experiments. Error bars show plus/minus SEM; asterisks indicate mean values different from Col‐0 mock treatment (one‐way ANOVA; Tukey–Kramer post‐hoc test, *P *<* *0.05). See Supporting Information Table S9 for source data and statistics.

To test whether HaRxL106 binding to RCD1 correlates with its defense‐suppressing activities, we generated a mutant variant of HaRxL106 that does not bind to RCD1. We used the Y2H system to compare RCD1 binding to full‐length HaRxL106, an HaRxL106 variant lacking the 56 C‐terminal amino acids (HaRxL106ΔC), with RCD1 binding to the C‐terminal 58 amino acids alone (HaRxL106‐Cterm58). In contrast to Mukhtar *et al*. (2011), we did not detect interaction between the two full‐length proteins by Y2H under our conditions. However, we found that the HaRxL106 C‐terminus interacts with RCD1 (Fig. 6a). Next, we tested which domains of RCD1 are required for this interaction. As shown in Fig. 6(a), the HaRxL106 C‐terminus interacted with a fragment spanning the WWE and PARP domains but did not bind to the isolated WWE, PARP, or RST domains. Notably, the RCD1 WWE–PARP construct also showed interaction with full‐length HaRxL106 protein (Fig. 6a). This suggests that HaRxL106 specifically binds to the RCD1 WWE–PARP domains via its C‐terminal region.

We next tested whether the HaRxL106 C‐terminal 58 amino acids are necessary for altered light and SA signaling in *Arabidopsis*. We transformed an *RFP:NLS:HaRxL106*Δ*C* construct lacking the C‐terminus of the effector into Col‐0. Because the *HaRxL106*Δ*C* construct also lacks the effector's NLS, this fusion protein carries an SV40 NLS to ensure efficient nuclear import (Wirthmueller *et al*., 2015). As controls, we generated transgenic RFP:HaRxL106 lines and lines expressing RFP fused to the 58 C‐terminal amino acids of HaRxL106 (RFP:HaRxL106‐Cterm58). All constructs were under control of the *35S* promoter, and we confirmed expression of the RFP fusion proteins by western blot (Fig. 6b). In comparison with Col‐0, transgenic lines expressing RFP‐tagged HaRxL106 developed longer petioles and a reduced leaf area (Fig. 6c). By contrast, RFP:NLS:HaRxL106ΔC lines were indistinguishable from wild‐type plants. RFP‐HaRxL106‐Cterm58 lines resembled transgenics expressing full‐length HaRxL106, suggesting that the C‐terminus of the effector is required and sufficient for attenuation of light signaling (Fig. 6c). We then tested resistance to *Hpa* Noco2 in these lines. In contrast to RFP:HaRxL106, the truncated RFP:NLS:HaRxL106ΔC protein failed to suppress defense (Fig. 6d; Table S8). Transgenic lines expressing RFP:HaRxL106‐Cterm58 were more susceptible to *Hpa* Noco2 than Col‐0 were, but less so than lines expressing the full‐length effector. Therefore, the C‐terminal 58 amino acids of HaRxL106 are required to attenuate defense signaling, and the same part of the effector protein alters plant growth responses to light. Similar to RFP:HaRxL106 lines, transgenics expressing the HaRxL106 C‐terminus responded with lower *PR1* transcript levels than wild‐type plants to SA spraying, whereas RFP:NLS:HaRxL106ΔC lines responded like wild‐type (Fig. 6e; Table S9).

### RCD1 is dispensable for resistance to *Hpa* but required for HaRxL106‐mediated suppression of defense

**Figure 7:**
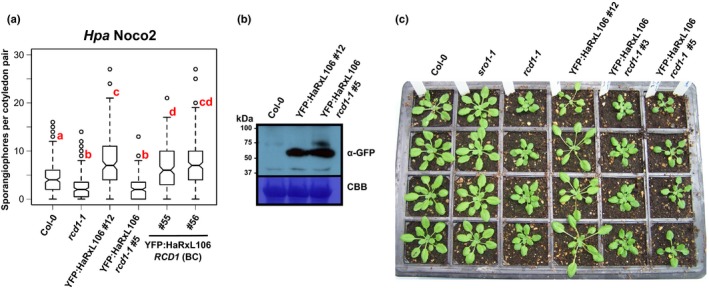
*Hyaloperonospora arabidopsidis* (*Hpa*) HaRxL106‐mediated suppression of immunity in *Arabidopsis thaliana* is abolished in *RADICAL‐INDUCED CELL DEATH1* (*RCD1*) loss‐of‐function mutants. (a) Resistance to the virulent *Hpa* isolate Noco2 in 10‐d‐old seedlings of Col‐0, *rcd1‐1*, the YFP:HaRxL106 line #12, a transgenic lines expressing YFP:HaRxL106 to comparable levels in the *rcd1‐1* background (#5), and two descendant lines of #5 in which RCD1 has been reintroduced by backcrossing to Col‐0 (#55 and #56). The plots show the number of conidiophores per cotyledon pair. Data from five independent biological experiments were pooled, horizontal bars show median, vertical box height represents interquartile range (IQR), and whisker range is 1.5× IQR. Circles represent data points beyond 1.5× IQR. Letters indicate differences between mean values (one‐way ANOVA; Tukey–Kramer post‐hoc test, *P *<* *0.05). See Supporting Information Table S10 for source data and statistics. (b) Western blot showing protein levels of YFP:HaRxL106 in line #12 (Col‐0) and line #5 (*rcd1‐1*). CBB, Coomassie brilliant blue stain. (c) Visual phenotype of 4‐wk‐old plants of Col‐0, *sro1‐1*,* rcd1‐1*, and lines expressing YFP:HaRxL106 in either Col‐0 or *rcd1‐1* backgrounds. The YFP:HaRxL106 fusion protein was not detectable by western blot in line YFP:HaRxL106 *rcd1‐1* #3.

To test whether *RCD1* contributes to resistance to *Hpa*, we infected the *rcd1‐1* mutant with *Hpa* Noco2. The *rcd1‐1* mutant showed enhanced resistance compared with Col‐0 (Fig. 7a; Table S10), which is consistent with a previous large‐scale *Hpa* phenotyping report (Weßling *et al*., 2014). This suggests that the lower level of defense gene expression before pathogen challenge in *rcd1‐1* does not compromise resistance against *Hpa*. Therefore, if RCD1 is a virulence target of HaRxL106, inhibition of RCD1's function(s) or signaling is unlikely to be responsible for the enhanced susceptibility induced by HaRxL106. We considered that HaRxL106 may manipulate RCD1 in a way that is not mimicked by complete loss of RCD1 function; for example, by converting RCD1 into a transcriptional co‐repressor. We compared susceptibility to *Hpa* Noco2 in the YFP:HaRxL106 line and a transgenic line expressing the same construct in an *rcd1‐1* mutant background. Although the YFP:HaRxL106 fusion protein accumulated to similar levels in both transgenic lines (Fig. 7b), the *rcd1‐1* mutation completely suppressed the enhanced susceptibility induced by YFP:HaRxL106. Reintroduction of functional *RCD1* by crossing restored HaRxL106 function (Fig. 7a; Table S10). Therefore, functional RCD1 protein is essential for suppression of defense by HaRxL106. Loss of *RCD1* function also attenuated the extent of petiole elongation in the YFP:HaRxL106 background (Fig. 7c).

### A crystal structure of RCD1's PARP domain suggests that RCD1‐type proteins do not function as canonical ADP‐ribosyl transferases

**Figure 8:**
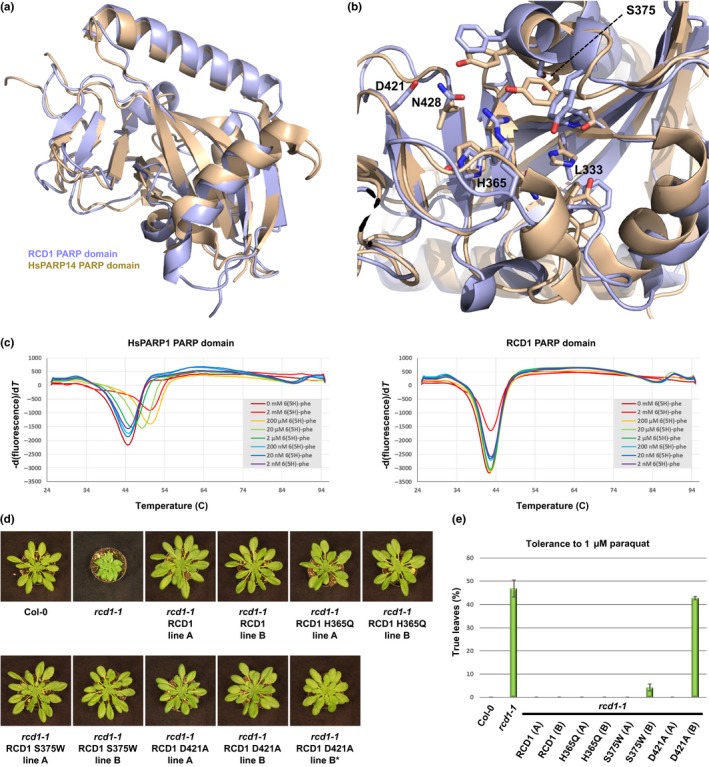
Mutations in *Arabidopsis thaliana* RADICAL‐INDUCED CELL DEATH1's (RCD1's) putative NAD^+^ binding pocket do not compromise RCD1 function in development and oxidative stress signaling. (a) Crystal structure of the RCD1 PARP domain (light blue, PDB code 5NGO) superimposed onto the structure of HsPARP14 (beige, PDB code 3SE2) (Wahlberg *et al*., 2012). For X‐ray data collection, refinement, and validation statistics, see Supporting Information Table S11. (b) Structural comparison of HsPARP14's NAD^+^ binding site (beige) with the corresponding area of the RCD1 PARP domain (light blue). RCD1 residues L333, H365, and N428 take the positions of the conserved H‐Y‐E triad in canonical PARPs. (c) Effect of the nonspecific PARP inhibitor 6(5*H*)‐phenanthridinone on the thermal stability of HsPARP1's PARP domain (left panel) and the RCD1 PARP domain (right panel). See Table S12 for source data from three independent experiments. (d) Morphological phenotypes of RCD1 site‐directed mutants with the indicated amino acid exchanges in the putative NAD^+^ binding site. Images are representative of > 10 plants per line. RCD1 D421A line B showed both plants that fully complemented and individuals that partially complemented (asterisk) the developmental phenotype of *rcd1‐1*. (e) Tolerance of Col‐0, *rcd1‐1*, and RCD1 site‐directed mutants to paraquat. Seeds were sown on Murashige‐Skoog plates containing 1 μm paraquat and the percentage of seedlings (*n *=* *48) that had developed true leaves after 20 d was determined. The plot shows the mean value of two independent biological experiments. Error bars show plus/minus SEM. See Table S13 for source data.

Our finding that suppression of defense by HaRxL106 is largely dependent on RCD1 and that the effector binds to RCD1's WWE–PARP domains prompted us to further investigate the molecular function(s) of RCD1. We reasoned that if RCD1 had PARP or a related transferase activity, HaRxL106 might manipulate this enzymatic function. We solved a crystal structure of the RCD1 PARP domain by X‐ray crystallography (PDB ID 5NGO; Table S11; Methods S1). The RCD1 PARP domain adopts a fold that is overall similar to mammalian PARP domains (Fig. 8a). However, the RCD1 PARP domain structure confirmed that the amino acid triad H‐Y‐E, constituting the active site of mammalian and plant PARPs, is not conserved in RCD1 (Kleine *et al*., 2008; Jaspers *et al*., 2010) (Fig. 8b). Nonconservation of the His and Tyr residues critical for NAD^+^ binding in canonical ADP‐ribosyl‐transferases suggests that the RCD1 PARP domain does not bind NAD^+^ and therefore is likely to lack canonical PARP activity. Consistent with this inference, the RCD1 PARP domain is not stabilized by 6(5*H*)‐phenanthridinone, an inhibitor of mammalian PARPs (Wahlberg *et al*., 2012), at elevated temperatures (Fig. 8c; Table S12). Conceivably, the cleft of RCD1 that corresponds to the catalytic center of active PARPs has evolved to bind other small compounds. To test whether this region of the protein is required for the biological function of RCD1, we designed three RCD1 mutant variants with single amino acid exchanges in the cleft region. When expressed under transcriptional control of 2.5 kb of the native *RCD1* promoter, all constructs complemented the developmental phenotype (Fig. 8d) and the enhanced paraquat tolerance of the *rcd1‐1* mutant (Fig. 8e; Table S13; Ahlfors *et al*., 2004). One transgenic line expressing RCD1 D421A did not complement the oxidative stress phenotype of *rcd1‐1* (Fig. 8e) and only partially complemented the developmental phenotype of *rcd1‐1* (compare lines B and B* in Fig. 8d). However, an independent transgenic line (A) expressing the same construct fully complemented both *rcd1‐1* phenotypes, suggesting that differences in expression levels might account for partial complementation in line B. Overall, our results suggest that the integrity of the presumed NAD^+^ binding cleft is not essential for RCD1's functions in plant development and signal transduction under oxidative stress conditions.

### HaRxL106 binds to the N‐terminal domains of RCD1 and SRO1 that mediate homo‐ and heterodimerization

**Figure 9:**
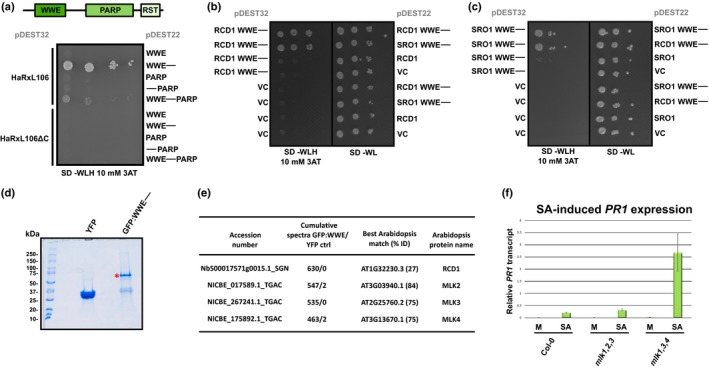
The N‐terminal WWE‐linker region of *Arabidopsis thaliana* RADICAL‐INDUCED CELL DEATH1 (RCD1) forms homo‐ and hetero‐oligomers and interacts with Mut9‐like kinases (MLKs). (a) Mapping protein–protein interactions between HaRxL106 and the indicated RCD1 domain(s) in the yeast‐two‐hybrid assay. The HaRxL106ΔC deletion construct does not bind RCD1 and is shown as a control. (b, c) Formation of homo‐ and hetero‐oligomers by RCD1's (b) and SIMILAR TO RCD ONE1's (SRO1's) (c) WWE‐linker region in the yeast‐two‐hybrid assay. VC, pDEST22, or pDEST32 vector control. (d) Representative sodium dodecyl sulfate polyacrylamide gel electrophoresis image of transiently expressed YFP and RCD1 GFP:WWE‐linker proteins immuno‐purified from *Nicotiana benthamiana*. The gel was stained with colloidal blue Coomassie. The red asterisk indicates the expected migration of the GFP:WWE‐linker fusion protein. (e) Selected proteins that were enriched in immunoprecipitates of the GFP:WWE‐linker fusion protein compared with the YFP control. For the full list, see Supporting Information Table S14. (f) Salicylic acid (SA)‐induced *PATHOGENESIS‐RELATED GENE 1* (*PR1*) gene expression in Col‐0 and the *mlk1,2,3* and *mlk1,3,4* triple mutants. Four‐week‐old plants were sprayed with 0.1 mM SA or a mock (M) solution and *PR1* expression levels were analyzed by quantitative real‐time PCR 8 h later. *PR1* expression levels were normalized by *ELONGATION FACTOR 1 ALPHA* (*EF1α*) expression. The plot shows the mean of *PR1/EF1α* expression from three independent biological experiments. Error bars show plus/minus SEM; asterisk indicates mean value different from Col‐0 SA treatment (one‐way ANOVA; Tukey–Kramer post‐hoc test, *P *<* *0.05). See Table S17 for source data and statistics.

Using the Y2H system, we further narrowed down the HaRxL106 binding site of RCD1 to an N‐terminal fragment encompassing the WWE domain and the linker region up to the beginning of the PARP domain (Fig. 9a). Deletion of the linker region resulted in loss of interaction with HaRxL106, suggesting that the WWE domain on its own is not sufficient for effector binding. The isolated PARP domain did not interact with HaRxL106, irrespective of whether or not we included the linker region (Fig. 9a). This suggests that the WWE‐linker region is required and sufficient for binding to HaRxL106. We found that the RCD1 WWE‐linker region interacts with itself and the corresponding region of SRO1 in Y2H assays, indicative of the formation of homo‐ and hetero‐oligomers (Fig. 9b). We obtained comparable results for the corresponding part of the SRO1 protein (Fig. 9c). These data suggest that the RCD1 and SRO1 WWE‐linker regions could mediate formation of RCD1/SRO1 oligomers.

### RCD1's WWE domain forms protein complexes with MLKs

Given that RCD1 does not have PARP activity, we further characterized RCD1 protein function(s) by screening for *in planta* interactors of RCD1. Attempts to immuno‐purify epitope‐tagged RCD1 protein in amounts sufficient for LC–MS/MS analysis of co‐purifying proteins from transient expression assays in *N. benthamiana* or stable *Arabidopsis* transgenics were not successful. We therefore resorted to screening for interactors of RCD1's WWE‐linker region following transient expression in *N. benthamiana*, as this part of the protein binds to HaRxL106 and is more stable (Fig. 9d). The predominant interactors were several importin‐α isoforms, full‐length RCD1‐type proteins and protein kinases with sequence homology to casein I kinases (Fig. 9e; Table S14). Identification of peptides from the PARP and RST domains in pulldown experiments of the WWE‐linker domain suggest that this domain forms homo‐ and hetero‐oligomers with endogenous RCD1‐type proteins in *N. benthamiana* (Fig. 9; Fig  S2). This is consistent with oligomer formation of the WWE‐linker regions in Y2H. A BLASTP search of the co‐purifying casein‐I‐related kinases against the *Arabidopsis* protein database (TAIR11) identified MLKs as likely orthologues (Fig. 9e). MLKs, also described as PHOTOREGULATORY PROTEIN KINASES, are nuclear‐localized Ser/Thr kinases that phosphorylate the photoreceptor cryptochrome 2 (CRY2) and PHYTOCHROME INTERACTING FACTOR 3 (PIF3) (Liu *et al*., 2017; Ni *et al*., 2017). In *Arabidopsis* and *Chlamydomonas*, phosphorylation of histone H3 Thr3 (H3T3ph) is another well‐characterized MLK phosphorylation site (Casas‐Mollano *et al*., 2008; Wang *et al*., 2015). We identified several phosphopeptides from the RCD1 WWE‐linker region (Fig. S3; Table S15). While most of these phosphorylated peptides were located in the GFP:WWE‐linker bait protein from *Arabidopsis*, we also detected two phosphopeptides from the WWE–PARP linker region of a co‐purifying *N. benthamiana* RCD1 orthologue (Figs S3, S4; Table S15), indicating that RCD1‐type proteins are phosphoproteins. We confirmed interactors identified in *N. benthamiana* and phosphorylation of the RCD1 WWE‐linker region in a single experiment using a stable transgenic *Arabidopsis* line expressing *35S*_*Pro*_ *:GFP:WWE‐linker* protein (Table S16; Figs S2, S3). Overall, our results show that MLKs interact with the RCD1N‐terminal domain in plant cells, suggesting a possible role of RCD1 and sequence‐related proteins in influencing covalent modifications of light‐regulatory components and/or histone tails.

MLKs have been previously reported to affect H3T3ph levels in response to osmotic and salt stress, and, like *rcd1* mutants, the *mlk1,2* double mutant is hypersensitive to sublethal concentrations of sodium chloride (Katiyar‐Agarwal *et al*., 2006; Wang *et al*., 2015). As MLKs and RCD1 form protein complexes in plant cells, and given that SA marker genes are expressed at lower levels in *rcd1* mutants, we asked whether MLKs also affect the transcriptional response to SA. We sprayed *mlk1,2,3* and *mlk1,3,4* triple mutants with SA and determined *PR1* transcript levels 8 h later (Fig. 9f; Table S17). While *PR1* levels in the *mlk1,2,3* triple mutant did not differ from wild‐type, the *mlk1,3,4* triple mutant consistently showed elevated *PR1* transcript levels in response to SA. However, under our growth conditions, the *mlk1,3,4* triple mutant did not show enhanced disease resistance upon infection with the adapted *Pst* DC3000 strain (Fig. S5; Table S18).

## Discussion

Several biotrophic pathogens evolved virulence mechanisms to counteract activation of SA‐dependent defense genes (Asai *et al*., 2014; Lewis *et al*., 2015). Apart from enzymatic conversion of SA precursors (Djamei *et al*., 2011; Liu *et al*., 2014b), effector‐mediated activation of JA signaling appears to be the main strategy of biotrophic pathogens to attenuate SA‐dependent defense (Zheng *et al*., 2012; Caillaud *et al*., 2013; Yang *et al*., 2017). Here, we show that ectopic expression of *Hpa* effector HaRxL106 suppresses both the expression of SA marker genes and the JA/ethylene marker gene *PDF1.2* in noninfected plants (Fig. 4; Table S6). This suggests that HaRxL106 manipulates SA signaling via a mechanism that does not rely on activation of JA signaling. The growth phenotype of HaRxL106‐expressing transgenic plants is consistent with constitutive shade avoidance, and conceivably HaRxL106 could suppress plant immunity by manipulating light signal transduction. However, we note that an effect on temperature sensing, brassinosteroids (BRs), or auxin levels could also underlie this phenotype. Several genes that are overexpressed in the HaRxL106 transgenic line are also altered in expression in constitutive BR signaling mutants (Table S6). However, unlike constitutive BR‐signaling mutants or plants with elevated BR levels that develop longer hypocotyls than wild‐type plants do in darkness (Choe *et al*., 2001; Jaillais *et al*., 2011; Gou *et al*., 2012), HaRxL106‐expressing lines do not show this phenotype (Fig. 1d; Table S2). Elevated temperatures promote auxin‐mediated hypocotyl elongation in the light (Gray *et al*., 1998). By contrast, etiolated seedlings of auxin‐overproducing lines develop shorter hypocotyls than wild‐type seedlings do (Zhao *et al*., 2001; Nishimura *et al*., 2014). Despite a trend for shorter hypocotyls in etiolated seedlings of HaRxL106‐expressing lines, the effect was small and not statistically significant in most experiments (Fig. 1d; Table S2). Notably, PIF transcription factors play a dual role in light and elevated‐temperature signaling (Koini *et al*., 2009; Leivar & Monte, 2014), and recent results suggest that phytochromes may act as light and temperature sensors (Jung *et al*., 2016; Legris *et al*., 2016). Given this apparent early conversion of signaling pathways for light and elevated temperature, dissecting how HaRxL106 promotes plant elongation growth in response to environmental signals requires a more detailed analysis.

We mapped the defense‐manipulating activity of HaRxL106 to a short C‐terminal part of the effector, which is essential for binding to RCD1 (Figs 6, 7). Notably, the effect of HaRxL106 on plant growth responses to light is mediated by the same region of the effector (Fig. 6). In *RCD1* loss of function mutants, HaRxL106‐mediated suppression of defense is abolished and HaRxL106‐induced petiole elongation is diminished (Fig. 7). These results suggest that RCD1 integrates both environmental signals and information from immune receptors, and that *Hpa* exploits this function of RCD1 to attenuate plant immunity. Notably, the growth and defense phenotypes of *rcd1* null mutants are opposite to those induced by ectopic expression of HaRxL106 (Fig. 7). This indicates that the effector manipulates RCD1 in a way that is not mimicked by *RCD1* loss of function alleles. RCD1 is essential for maintaining the basal expression levels of SA‐inducible defense genes in noninfected plants, but it is dispensable for transcriptional activation of these defense genes upon pathogen infection (Fig. 4; Table S6). One conceivable mode of HaRxL106 function is that effector binding to RCD1 converts the latter into a transcriptional co‐repressor of defense genes (Fig. S6). However, this hypothesis cannot easily be tested without a better understanding of the molecular function of both proteins.

HaRxL106 is predicted to have an α‐helical WY structure, a fold that likely evolved as a versatile building module of oomycete effectors and can mediate different molecular functions in fusion with small peptides or other domains (Boutemy *et al*., 2011; Maqbool *et al*., 2016). The WY domain in HaRxL106 might function as a scaffold that stabilizes and/or presents the C‐terminal peptide that is essential for suppression of plant immunity. In accordance with this model, expressing a fusion of the C‐terminal 58 amino acids of HaRxL106 to RFP is sufficient to alter plant growth responses to light and attenuate defense (Fig. 6). Notably, manipulation of selective autophagy by the host‐targeted *P. infestans* effector PexRD54 is also based on a disordered C‐terminal peptide that is stabilized by five tandem WY domains (Dagdas *et al*., 2016; Maqbool *et al*., 2016).

RCD1, and sequence‐related proteins from *Arabidopsis* and rice (*Oryza sativa*), bind transcription factors via their C‐terminal RST domains. By contrast, the functions of RCD1's N‐terminal WWE and central PARP domains have not been characterized. Although an RCD orthologue from wheat shows PARP activity (Liu *et al*., 2014a), our structural analysis suggests that *Arabidopsis* RCD1 is unlikely to be enzymatically active. This is consistent with the previously reported finding that recombinantly expressed GST:RCD1 does not ADP‐ribosylate histones or itself (Jaspers *et al*., 2010). Our crystal structure of the RCD1 PARP domain provides first insights into plant PARP domains, and we identified several molecular differences between RCD1's PARP domain and the catalytic domain of mammalian PARPs. Nonconservation of the H‐Y‐E triad (Fig. 8b) and our finding that RCD1 retains its biological function when amino acids within the presumed active site are mutated (Fig. 8d,e) suggest that the protein does not have PARP activity. Given that the N‐ and C‐terminal domains of RCD1 mediate protein–protein interactions, the PARP domain of RCD1 might act as a scaffold bridging and/or coordinating the action of the terminal protein interaction domains.

RCD1's WWE domain and the linker region up to the PARP domain are essential for binding to HaRxL106 (Fig. 9a). The WWE domains of RCD1 and its paralogue SRO1 can also form homo‐ and hetero‐oligomers in Y2H (Fig. 9b,c). The WWE domain is a conserved iso‐ADP‐ribose binding domain, but it is not known whether plant WWE domains bind poly(ADP‐ribose) chains (He *et al*., 2012; Wang *et al*., 2012). Conceivably, HaRxL106 binding to RCD1's WWE domain could interfere with ADP‐ribose binding if this biological function is conserved in plants. An alternative, but not mutually exclusive, scenario is that RCD1's WWE domain is an interaction module for other proteins. Here, we identified kinases from the MLK group as novel interactors of RCD1's WWE‐linker domain. Consistent with complex formation between the N‐terminal domain of RCD1 and MLKs, we identified several phosphorylation sites in RCD1's linker region (Fig. S3; Table S15). The interaction between MLKs and the RCD1 N‐terminus implies that phosphorylation of the linker region might be mediated by MLKs, but our data do not rule out alternative kinases. The ~ 90 amino acid linker region between the RCD1 WWE and PARP domains is predicted to be disordered (Ishida & Kinoshita, 2007; Kragelund *et al*., 2012), but it is conceivable that phosphorylation or binding of interacting proteins induces a specific fold in this region (Wright & Dyson, 2009; Bah *et al*., 2015). If the RCD1 PARP domain acts as a scaffold, reversible phosphorylation of residues in the linker region between WWE and PARP domains could regulate the cooperation of these two domains. Although the molecular functions of RCD1 remain poorly characterized, its localization to the nucleus and interaction with transcription factors point to a role as a transcriptional co‐regulator. Consistent with such a role of RCD1 is our finding that HaRxL106 interferes with SA signaling at the level of transcription (Fig. 3).

MLKs are recruited to the evening complex in a phyB‐dependent manner and have previously reported functions in light signaling, circadian rhythm, and abiotic stress responses (Casas‐Mollano *et al*., 2008; Wang *et al*., 2015; Huang *et al*., 2016). Notably, bluelight‐dependent phosphorylation and subsequent proteasomal degradation of the photoreceptor CRY2 requires MLKs (Liu *et al*., 2017). MLKs also mediate red‐light‐induced phosphorylation of PIF3, form a red‐light‐induced ternary complex with phyB and PIF3, and phyB protein levels are elevated in *mlk* multiple mutants (Ni *et al*., 2017). Therefore, MLKs appear to constitute a phosphoregulatory signaling node at the level of photoreceptors and associating transcription factors. RCD1 also interacts with PIF transcription factors, and *rcd1* mutants show reduced hypocotyl elongation under red and blue light (Jaspers *et al*., 2009; Salazar & Neuhaus, 2010). Based on these results, we speculate that HaRxL106 targets RCD1 or RCD1‐containing protein complexes to manipulate light and salicylate signaling. An *mlk1,3,4* triple mutant shows elevated *PR1* transcript levels following SA application when compared with wild‐type plants (Fig. 9f). This suggests that MLKs influence transcriptional mechanisms required for fine‐tuning the amplitude of SA‐induced *PR1* expression. H3T3 is a well‐characterized phosphorylation site of MLKs in *Chlamydomonas* and *Arabidopsis*. In mammalian cells, repressive H3T3ph and activating tri‐methylation of the adjacent K4 form a molecular switch that directly affects transcription factor II D binding, thereby regulating gene expression throughout the cell cycle (Varier *et al*., 2010).

In summary, our analysis of RCD1 as a target of the *Arabidopsis* downy mildew effector HaRxL106 suggests that RCD1 and MLKs form a nuclear hub that integrates and relays information from signaling pathways sensing environmental cues and pathogen infection. We further show that the *Arabidopsis* downy mildew pathogen *Hpa* manipulates this signaling node to prevent activation of SA signaling.

## Author contributions

L.W., S.A., G.R., G.F., D.S.K., M.W., J.K., M.J.B. and J.D.G.J. designed the research; L.W., S.A., G.R., J.S., G.F., D.S.K., R.L., P.J. and M.W. performed the research; L.W., S.A., G.R., J.S., G.F., D.S.K., M.W., D.M., F.L.H.M., M.J.B. and J.D.G.J. carried out data analysis, collection, or interpretation; L.W., S.A., G.R., J.S., G.F., D.S.K., M.W., J.K., D.M., F.L.H.M., M.J.B. and J.D.G.J. wrote the manuscript. All authors have edited and approved the submitted version of the manuscript. The authors declare that they have neither financial nor nonfinancial competing interests. S.A. and G.R. contributed equally to this work.

## Supporting information

Please note: Wiley Blackwell are not responsible for the content or functionality of any Supporting Information supplied by the authors. Any queries (other than missing material) should be directed to the *New Phytologist* Central Office.

**Fig. S1** Transcriptome profiling of *Arabidopsis thaliana Pro*_*35S*_ *:HS:HaRxL106* line #2 and knock‐out mutants of the *Hyaloperonospora arabidopsidis* HaRxL106‐interacting proteins MOS6, ASIL1 and RCD1.
**Fig. S2** Sequence coverage of *Nicotiana benthamiana* RCD1, *Arabidopsis thaliana* RCD1 and *A. thaliana* SRO1 identified by peptide fingerprinting in immunoprecipitation experiments of the RCD1 GFP:WWE‐linker fusion protein.
**Fig. S3** Phosphopeptides identified in the *Arabidopsis thaliana* RCD1 GFP:WWE‐linker fusion protein and a co‐purifying *Nicotiana benthamiana* RCD1 ortholog.
**Fig. S4** Three MS spectra supporting the two identified phosphopeptides of the *Nicotiana benthamiana* RCD1 ortholog.
**Fig. S5** Growth of *Pseudomonas syringae* pv. *tomato* (*Pst*) DC3000 in leaves of *Arabidopsis thaliana* Col‐0, the two *mlk* triple mutants and the *sid2‐1* mutant.
**Fig. S6** Model of *Hyaloperonospora arabidopsidis* HaRxL106‐mediated manipulation of defense gene expression in *Arabidopsis thaliana*.

**Table S1** Quantification of *Hyaloperonospora arabidopsidis* Noco2 conidiophores on adult leaves of *Arabidopsis thaliana* and statistical analysis based on two independent biological experiments
**Table S2** Quantification of *Arabidopsis thaliana* seedling hypocotyl length and statistical analysis based on three independent biological experiments
**Table S3** Quantification of *Arabidopsis thaliana* relative *PR1* transcript levels by qRT‐PCR and statistical analysis based on three independent biological experiments
**Table S4** Quantification of SA levels in *Arabidopsis thaliana* and statistical analysis based on three independent biological experiments
**Table S5** Quantification of *Arabidopsis thaliana* relative *PR1* transcript levels by qRT‐PCR and statistical analysis based on three independent biological experiments
**Table S7** Quantification of *Arabidopsis thaliana* relative *PR1* transcript levels by qRT‐PCR and statistical analysis based on five independent biologicals experiments
**Table S8** Quantification of *Hyaloperonospora arabidopsidis* Noco2 conidiophores on cotyledons of *Arabidopsis thaliana* and statistical analysis based on three independent biological experiments
**Table S9** Quantification of *Arabidopsis thaliana* relative *PR1* transcript levels by qRT‐PCR and statistical analysis based on three independent biological experiments
**Table S10** Quantification of *Hyaloperonospora arabidopsidis* Noco2 conidiophores on cotyledons of *Arabidopsis thaliana* and statistical analysis based on five independent biological experiments
**Table S13** Quantification of Paraquat tolerance in *Arabidopsis thaliana* Col‐0, *rcd1‐1* and transgenic lines expressing *RCD1* mutant variants in the *rcd1‐1* background based on two independent experiments
**Table S16** List of proteins identified in the immunoprecipitate of the *Arabidopsis thaliana* RCD1 WWE‐linker bait protein expressed in *A. thaliana* in a single experiment
**Table S17** Quantification of *Arabidopsis thaliana* relative *PR1* transcript levels by qRT‐PCR and statistical analysis based on three independent biological experiments
**Table S18** Quantification of *Pseudomonas syringae* pv. *tomato* DC3000 growth in *Arabidopsis thaliana* Col‐0 and *mlk* triple mutants

**Table S6** Expression values of *Arabidopsis thaliana* SA and JA marker genes shown in Fig. 4 and detailed analysis of differentially expressed genes in *Pro*_*35S*_ *:HS:HaRxL106* line #2 and *rcd1‐1* using GO term annotation and analysis of cis‐regulatory elements

**Table S11** X‐ray data collection, refinement, and validation statistics for the *Arabidopsis thaliana* RCD1 PARP domain structure

**Table S12** Effect of different 6(5H)‐phenanthridinone concentrations on the thermal stability of the *Arabidopsis thaliana* RCD1 and *Homo sapiens* PARP1 PARP domains as determined by thermal shift assays and monitored in three independent experiments

**Table S14** List of proteins identified in immunoprecipitates of the *Arabidopsis thaliana* RCD1 WWE‐linker bait protein expressed in *Nicotiana benthamiana*

**Table S15** Phosphopeptides identified in immunoprecipitates of the *Arabidopsis thaliana* RCD1 WWE‐linker bait protein expressed in *Nicotiana benthamiana* in three independent biological experiments

**Methods S1** Methods for *Arabidopsis thaliana* Paraquat treatment and SA quantification, nucleic acid extraction and quantification, protein extraction and purification, microscopy, protein crystallography and protein mass spectrometry.
