# Supplementary material for: Arabidopsis downy mildew effector HaRxL106 suppresses plant immunity by binding to RADICAL‐INDUCED CELL DEATH1
Source: New Phytol. 2018 Aug 29;220(1):232–48. doi: 10.1111/nph.15277 (PMC6175486; doi:10.1111/nph.15277)
Supplement: Supplementary file 1 — Fig. S1 Transcriptome profiling of Arabidopsis thaliana Pro 35S :HS:HaRxL106 line #2 and knock‐out mutants of the Hyaloperonospora arabidopsidis HaRxL106‐interacting proteins MOS6, ASIL1 and RCD1. Fig. S2 Sequence coverage of Nicotiana benthamiana RCD1, Arabidopsis thaliana RCD1 and A. thaliana SRO1 identified by peptide fingerprinting in immunoprecipitation experiments of the RCD1 GFP:WWE‐linker fusion protein. Fig. S3 Phosphopeptides identified in the Arabidopsis thaliana RCD1 GFP:WWE‐linker fusion protein and a co‐purifying Nicotiana benthamiana RCD1 ortholog. Fig. S4 Three MS spectra supporting the two identified phosphopeptides of the Nicotiana benthamiana RCD1 ortholog. Fig. S5 Growth of Pseudomonas syringae pv. tomato (Pst) DC3000 in leaves of Arabidopsis thaliana Col‐0, the two mlk triple mutants and the sid2‐1 mutant. Fig. S6 Model of Hyaloperonospora arabidopsidis HaRxL106‐mediated manipulation of defense gene expression in Arabidopsis thaliana. [file NPH-220-232-s001.pdf]

***New Phytologist* Supporting Information Figs S1-S6**

Article title: *Arabidopsis* Downy Mildew effector HaRxL106 suppresses plant immunity by binding to RADICAL-INDUCED CELL DEATH1

Authors: Lennart Wirthmueller, Shuta Asai, Ghanasyam Rallapalli, Jan Sklenar, Georgina Fabro, Dae Sung Kim, Ruth Lintermann, Pinja Jaspers, Michael Wrzaczek, Jaakko Kangasjärvi, Daniel MacLean, Frank L. H. Menke, Mark J. Banfield and Jonathan D. G. Jones

Article acceptance date: 09 May 2018

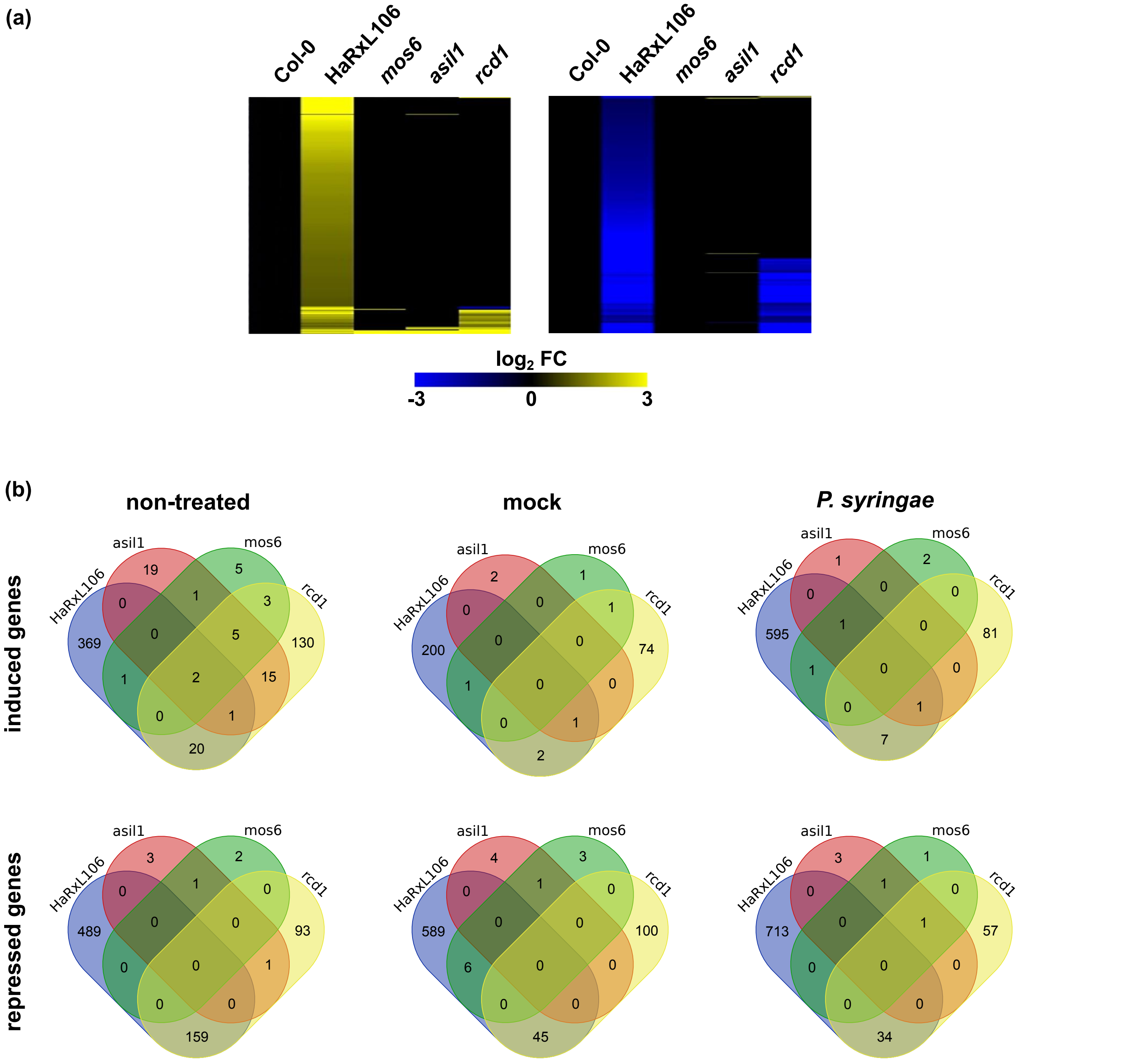

Figure S1. Transcriptome profiling of *Arabidopsis thalianaPro*$_{35S}$*:HS:HaRxL106* line #2 and knock-out mutants of the *Hyaloperonospora arabidopsidis* HaRxL106-interacting proteins MOS6, ASIL1 and RCD1. **(a)** Comparison of transcriptome changes induced by HaRxL106 overexpression and loss of function alleles of *mos6*, *asil1*, and *rcd1* under non-treated conditions. Up-regulated genes in yellow, repressed genes in blue, FC = fold change. Only those genes that differ in transcript levels from Col-0 in at least one of the other genotypes are shown. Significance cut-off is >2-fold change in expression and FDR<0.001. See Table S6 for a more detailed analysis of the RNA-seq data. **(b)** Overlap of genes that are induced or repressed in HS:HaRxL106 compared to Col-0 and the transcriptome profiles of *mos6*, *asil1* and *rcd1*. Venn diagrams were prepared using http://bioinformatics.psb.ugent.be/webtools/Venn/.

N. benthamiana

NbS00017571g0015.1_SGN (100%), 61,227.4 Da
Poly polymerase catalytic domain containing protein expressed
23 exclusive unique peptides, 26 exclusive unique spectra, 40 total spectra, 298/549 amino acids (54% coverag

```
MDLNCKVLKF MMTKTPADSI TPLKIAGDQN NAVSPVIDGN KLKIQVPVTR
KIVGGEVDVV DKKKVKIEVP IPRELLFPSA ETASCTDHAQ LLVQNYGNFK
KSGKPVRFMF YKDGSWVNFE KNVMDVMISG FVSGKPMIDV EMEGLKCLFD
FYRMLEIDMD TGKEHSISWI DVNGKCFFPK VFIDSSENSD DKNQEIEASN
VNGKFSSENP KIEIEIRISD DNSDKEVNNS GEELKLGKRK RGSEENEVEE
KGERSSSNAK ERRVIAATEL HSPRWPKARS LREEEKGYQM VKGLLLSGLR
TVDPAVTVTS IHQCVRTGPL EKARLEVFQT NMEIIKRARG GNLDVVYAWY
GTSAKNVEII LRHGFGMASV VHGSNAHGVG VYLSPLRQPQ NSAMMSEVDE
YGEKHIVLCR VILGKLEKVG LGSQQRYPSS VDFDTGVDDL TNPKWYVVWS
ANMNTHILPE CIVSYKSGRH MSGQANGASS MKWAPHASNA MGTLVSKLST
LLPPPKVQEL QSLYGSYQEG KLGKEVFMRQ LRSVVGDELL RSTILEIRG
```

A. thaliana

RCD1

AT1G32230.2 (100%), 65,569.5 Da
| Symbols: RCD1, CEO, CEO1, ATP8, AtRCD1 | WWE protein-protein interaction domain protein family | chr1:116
22 exclusive unique peptides, 30 exclusive unique spectra, 65 total spectra, 441/588 amino acids (75% coverag

```
MEAKIVKVLD SSRCEDGFGK KRKRAASYAA YVTGVSCAKL QNVPPPNGQC
QIPDKRRRLE GENKLSAYEN RSGKALVRYY TYFKKTGIAK RVMMYENGEW
NDLPEHVICA IQNELEEKSA AIEFKLCGHS FILDFLHMQR LDMETGAKTP
LAWIDNAGKC FFPEIYESDE RTNYCHHKCV EDPKQNAPHD IKLRLEIDVN
GGETPRLNLE ECSDESGDNM MDDVPLAQRS SNEHYDEATE DSCSRKLEAA
VSKWDETDAI VVSGAKLTGS EVLDKDAVKK MFAVGTASLG HVPVLDVGRF
SSEIAEARLA LFQKQVEITK KHRGDANVRY AWLPAKREVL SAVMMQGLGV
GGAFIRKSIY GVGIHLTAAD CPYFSARYCD VDENGVRYMV LCRVIMGNME
LLRGDKAQFF SGGEEYDNGV DDIESPKNYI VWNINMNTHI FPEFVVRFKL
SNLPNAEGNL IAKRDNSGVT LEGPKDLPPQ LESNGARGSG SANSVGSSTT
RPKSPWMPFP TLFAAISHKV AENDMLLINA DYQQLRDKKM TRAEFVRKLR
```

SRO1

AT2G35510.1 (100%), 64,138.8 Da
| Symbols: SRO1 | similar to RCD one 1 | chr2:14916898-14919198 REVERSE LENGTH=568
10 exclusive unique peptides, 12 exclusive unique spectra, 19 total spectra, 123/568 amino acids (22% coverag

```
MEAKIVKVSD SSYKDGLGKK RKHPGNYTPY DSGRSYAKLQ WVLSPNSSTQ
KLEKRRNLDG ENKVIVSENH VEKSLVRYFS YYKKTGVPKR VMFHENGEWI
DLPDHILCDI RNDLEAKRAT IEFNWCGRHF LLDFLHMYRL DLETGVKTQL
AWIDIAGKCF FPETFDTLER DGCHHIRGED PEQHDQREIK LHIEIDVNSG
ELPRLNLNVV TDESGDNMDD FQAVQRSSNG PNDEASEDSC SRELDDAVEK
WDKTETDRFS GVKPAEEELD KDAVKQMFAL GAATLGHVES LDVYQFSSEI
AKARLSLFQK QADITKKHRG DANIRYAWVP AKKEVLSAVM MHGLGVGGAF
IKKSMYGVGV HAANCPYFSA RYCDIDDNGV RHMVLCRVIM GNMEPLRGDN
TQYFTGGEEY DNGVDDVESP KHYLIWNMNM NTHIYPEFVV SFKLSIPNAE
GNILPTTQSR HESSGLTLEG PKGSPSNEPG RVSNGGSGSE KNSSSSRRPR
SPIMPFPLLF KAISSKIARK DMDLIIAGYQ ELREKKVSRK EFYKTLSMIV
```

Figure S2. Sequence coverage of *Nicotiana benthamiana* RCD1, *Arabidopsis thaliana* RCD1 and *A. thaliana* SRO1 identified by peptide fingerprinting in immunoprecipitationexperiments of the *Arabidopsis* RCD1 GFP:WWE-linker fusion protein. The identified tryptic peptides are shown in yellow. Green color indicates modified peptides (S/T/Y possible phosphorylation sites; M artificial oxidation or C carbamidomethylation).

Phosphopeptides from the GFP:WWE-linker bait expressed in *N. benthamiana*

MVSKGEELFTGVVPILVELDGDVNGHKFSVSGEGEGDATYGKLTLKFICTTGKLPVPWPTLVTTLT
YGVQCFSRYPDHMKQHDFFKSAMPEGYVQERTIFFKDDGNYKTRAEVKFEGDTLVNRIELKGIDF
KEDGNILGHKLEYNYNSHNVYIMADKQKNGIKVNFKIRHNIEDGSVQLADHYQQNTPIGDGPVLL
PDNHYLSTQSALSKDPNEKRDHMVLLEFVTAAGITLGMDELYKDITSLYKKAGSAAAPFTMEAKIV
KVLDSSRCEDGFGKKRKRAASYAAYVTGVSCAKLQNVPPPNGQCQIPDKRRRLEGENKLSAYEN
RSGKALVRYYTYFKKTGIAKRVMMYENGEWNDLPEHVICAIQNELEEKSAAIEFKLCGHSFILDFLH
MQRLDMETGAKTPLAWIDNAGKCFFPEIYESDERTNYCHHKCVEDPKQNAPHDIKLRLEIDVNGG
ETPRLNLEECSDESGDNMMDDVPLAQRSSNEHYDEATEDSCSRKLEAAVSKWDETDAIVVSGA

Phosphopeptides from the GFP:WWE-linker bait expressed in *A. thaliana*

MVSKGEELFTGVVPILVELDGDVNGHKFSVSGEGEGDATYGKLTLKFICTTGKLPVPWPTLVTTLT
YGVQCFSRYPDHMKQHDFFKSAMPEGYVQERTIFFKDDGNYKTRAEVKFEGDTLVNRIELKGIDF
KEDGNILGHKLEYNYNSHNVYIMADKQKNGIKVNFKIRHNIEDGSVQLADHYQQNTPIGDGPVLL
PDNHYLSTQSALSKDPNEKRDHMVLLEFVTAAGITLGMDELYKDITSLYKKAGSAAAPFTMEAKIV
KVLDSSRCEDGFGKKRKRAASYAAYVTGVSCAKLQNVPPPNGQCQIPDKRRRLEGENKLSAYEN
RSGKALVRYYTYFKKTGIAKRVMMYENGEWNDLPEHVICAIQNELEEKSAAIEFKLCGHSFILDFLH
MQRLDMETGAKTPLAWIDNAGKCFFPEIYESDERTNYCHHKCVEDPKQNAPHDIKLRLEIDVNGG
ETPRLNLEECSDESGDNMMDDVPLAQRSSNEHYDEATEDSCSRKLEAAVSKWDETDAIVVSGA

Phosphopeptides from *N. benthamiana* NICBE_210898.1_TGAC (Inactive poly [ADP-ribose] polymerase RCD1)

MDLNCKVLKFMMTKTPADSITPLKIAGDQNNAVSPVIDGNKLKIQVPVTRKIVGGEVDVVDKKKVKI
EVPIPRELLFPSAETASCTDHAQLLVQNYGNFKKSGKPVRFMFYKDGSWVNFEKNVMDVMISGFVS
GKPMIDVEMEGLKCLFDFYRMLEIDMDTGKEHSISWIDVNGKCFFPKVFIDSSENSDDKNQEIEASN
VNGKFSSENPKIEIEIRISDDNSDKEVNNSGEELKLGKRKRGSEENEVEEKGERSSSNAKERRVIAAT
ELHSPRWPKARSLREEEKGYQMVKGLLLSGLRTVDPAVTVTSIHQCVRTGPLEKARLEVFQTNMEII
KRARGGNLDVVYAWYGTSAKNVEIILRHGFGMASVVHGSNAHGVGVYLSPLRQPQNSAMMSEVD
EYGEKHIVLCRVILGKLEKVGLGSQQRYPSSVDFDTGVDDLTNPKWYVVWSANMNTHILPECIVSY
KSGRHMSGQANGASSMKWAPHASNAMGTLVSKLSTLLPPPKVQELQSLYGSYQEGKLGKEVFMR
QLRSVVGDELLRSTILEIRG

Figure S3. Phosphopeptides identified in the *Arabidopsis thaliana* RCD1 GFP:WWE-linker fusion protein and a co-purifying *Nicotiana benthamiana* RCD1 ortholog. The GFP sequence is indicated in green and phosphopeptides are shown in red.

MS spectra of phosphopeptides from *N. benthamiana* NICBE_210898.1_TGAC (Inactive poly [ADP-ribose] polymerase RCD1)

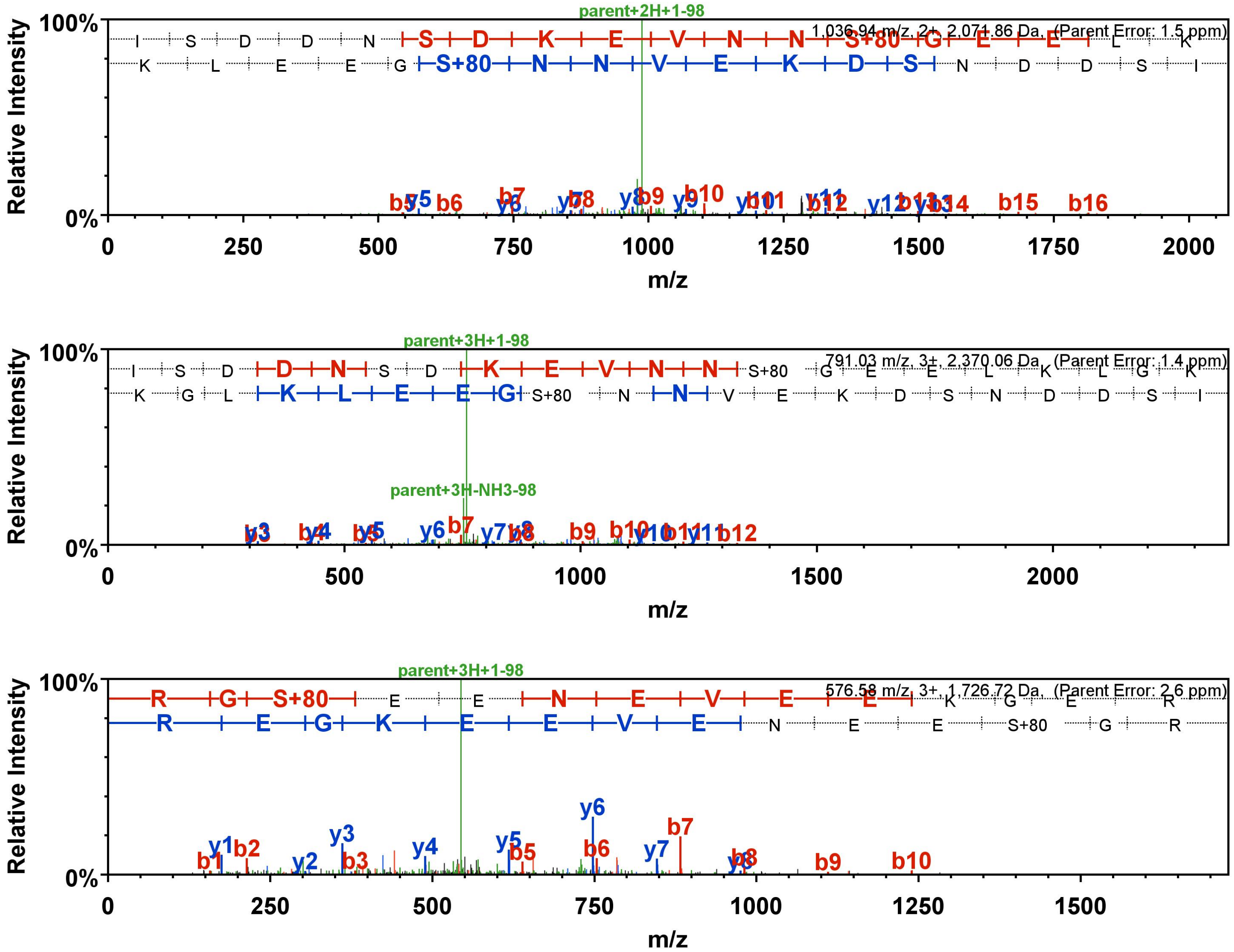

Figure S4. Three MS spectra supporting the two identified phosphopeptides of the *Nicotiana benthamiana* RCD1 ortholog.

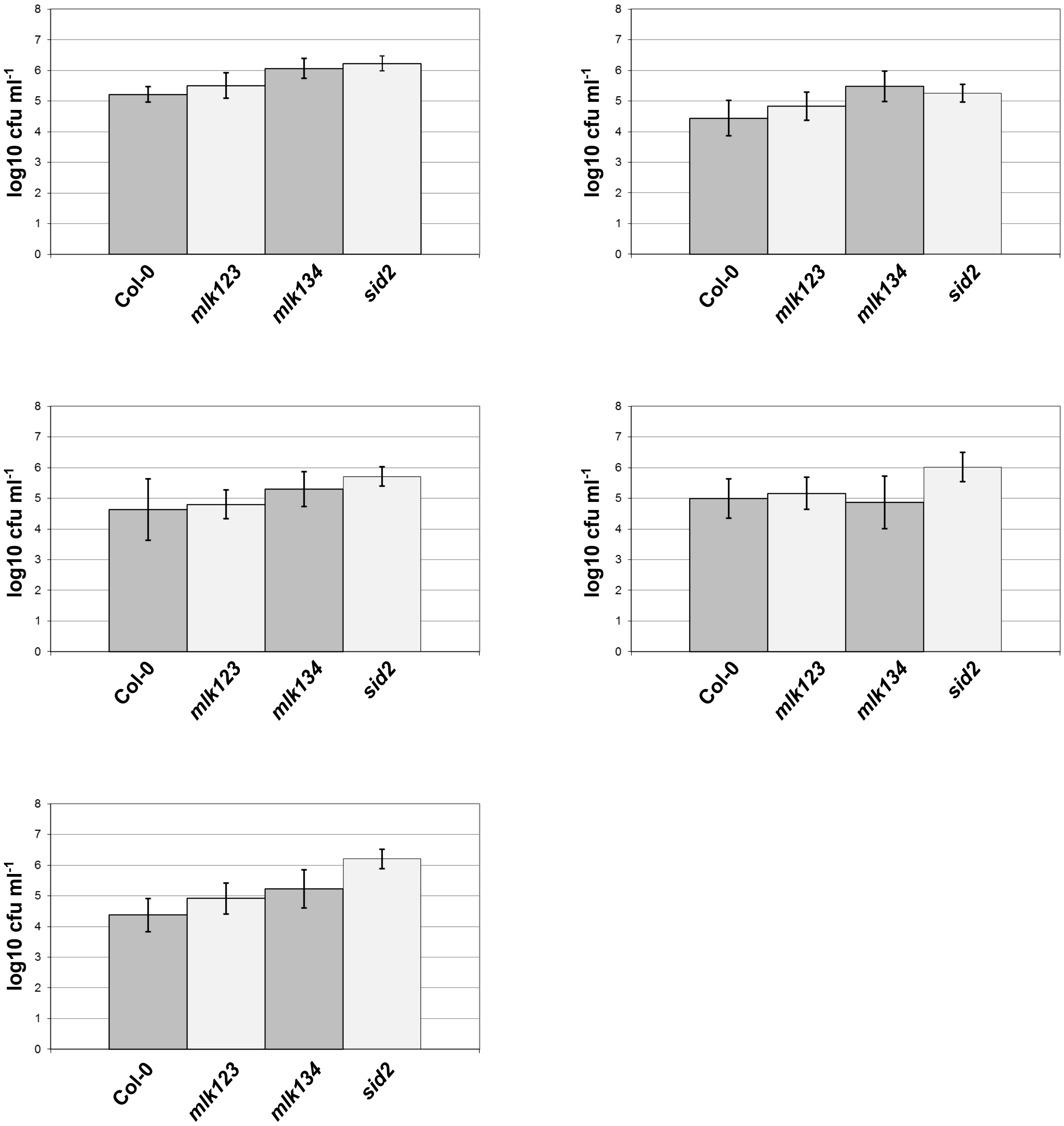

Figure S5. Growth of *Pseudomonas syringae* pv. *tomato* (*Pst*) DC3000 in leaves of *Arabidopsis thaliana* Col-0, the two *mlk* triple mutants and the *sid2-1* mutant. Rosette leaves of four-wk-old plants were syringe-infiltrated with $5x10^4$ cfu ml^{-1} of *Pst* DC3000 and bacterial titers were determined 3 d post infiltration by dilution plating on selective media. Five independent biological experiments are shown. Error bars denote STD. For source data and statistics see Table S18.

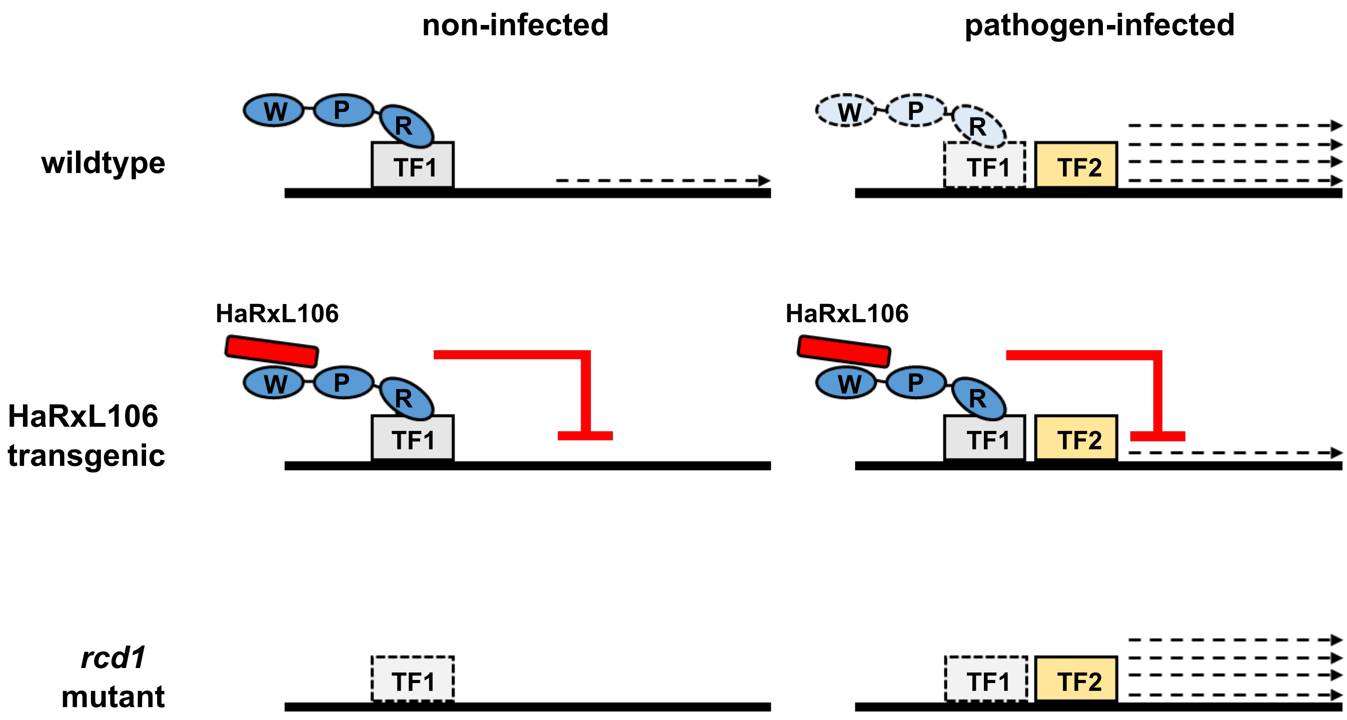

Figure S6. Model of *Hyaloperonospora arabidopsidis* HaRxL106-mediated manipulation of defense gene expression in *Arabidopsis thaliana*. In non-infected plants RCD1 acts as a co-transcriptional activator in complex with transcription factors (TF1) to maintain basal expression levels of SA-responsive defense genes. Upon pathogen infection RCD1 becomes dispensable for defense gene expression, conceivably because other defense-induced transcription factors (TF2) take over and mediate strong activation of defense gene expression. *In planta* expressed HaRxL106 binds to RCD1 and converts it into a co-transcriptional repressor thereby attenuating defense gene expression in non-infected and infected tissue. The model is based on the hypothesis that RCD1/TF complexes directly bind to defense gene promoters. Alternatively, the primary outcome of HaRxL106-mediated manipulation of RCD1 might be activation of the shade avoidance response, which in turn suppresses transcription of defense genes (de Wit *et al*. 2013). W, P, R = WWE, PARP and RST domains of RCD1. Dashed lines indicate inactive proteins or proteins that under the respective conditions do not contribute to transcriptional regulation of defense gene expression.

SI References

de Wit M, Spoel SH, Sanchez-Perez GF, Gommers CMM, Pieterse CMJ, Voesenek LACJ, Pierik R. **2013**. Perception of low red:far-red ratio compromises both salicylic acid- and jasmonic acid-dependent pathogen defences in *Arabidopsis*. *The Plant Journal: For Cell and Molecular Biology* **75**: 90–103.
