# Supplementary material for: Arabidopsis downy mildew effector HaRxL106 suppresses plant immunity by binding to RADICAL‐INDUCED CELL DEATH1
Source: New Phytol. 2018 Aug 29;220(1):232–48. doi: 10.1111/nph.15277 (PMC6175486; doi:10.1111/nph.15277)
Supplement: Supplementary file 4 — Table S11 X‐ray data collection, refinement, and validation statistics for the Arabidopsis thaliana RCD1 PARP domain structure [file NPH-220-232-s004.pdf]

***New Phytologist* Supporting Information Table S11**

Article title: *Arabidopsis* Downy Mildew effector HaRxL106 suppresses plant immunity by binding to RADICAL-INDUCED CELL DEATH1

Authors: Lennart Wirthmueller, Shuta Asai, Ghanasyam Rallapalli, Jan Sklenar, Georgina Fabro, Dae Sung Kim, Ruth Lintermann, Pinja Jaspers, Michael Wrzaczek, Jaakko Kangasjärvi, Daniel MacLean, Frank L. H. Menke, Mark J. Banfield and Jonathan D. G. Jones

Article acceptance date: 09 May 2018

Table S11 X-ray data collection, refinement, and validation statistics for the *Arabidopsis thaliana* RCD1 PARP domain structure.

Data collection	native	SeMet
Beamline	Diamond Light Source-i04	Diamond Light Source-i24
Wavelength (Å)	0.920	0.978
Space group	H 3 2	H 3 2
Unit cell parameters		
a, b, c (Å)	129.95, 129.95, 468.38	132.38, 132.38, 470.34
Unique reflections*	52927 (4530)	22387 (4541)
Resolution (Å)*	55.87 – 2.50 (2.58 – 2.50)	111.38 – 3.40 (3.67 – 3.40)
R_{merge} (%)*, #	0.117 (1.019)	0.236 (0.911)
I/σ(I)*, #	18.8 (5.2)	12.8 (5.6)
half-set correlation CC(1/2) *, #	0.999 (0.973)	0.998 (0.995)
Completeness (%)*, #	99.7 (99.9)	100 (100)
Anomalous completeness (%)*, #		100 (100)
Multiplicity*, #	30.8 (31.5)	23.8 (24.2)
Anomalous multiplicity*, #		12.4 (12.4)
DelAnom correlation between half-sets*, #		0.736 (0.095)
Mid-Slope of Anom Normal Probability		1.394

Refinement	
Resolution	55.88 – 2.50 (2.58 – 2.50)
R_{work} (%)[*, &]	0.2382 (0.3035)
R_{free} (%)[*, &]	0.2585 (0.3339)
Number of atoms	10837
B-factors (Å^2)[+]	62.2
rmsd	
Bond length (Å)[&]	0.012
Bond angles (º)[&]	1.440
Ramachandran favored (%)[+]	98.0
Ramachandran outliers (%)[+]	0.0
Overall MolProbity Score / percentile[+]	1.48 / 99th

[*]Values in parenthesis correspond to the highest resolution bin.

[#]Values reported by AIMLESS (Evans and Murshudov, 2013).

[&]Values reported by REFMAC (v5.8.0158) (Murshudov *et al.*, 2011).

[+]Values reported by MOLPROBITY (Chen *et al.*, 2010).

SI References

Chen VB, Arendall WB, Headd JJ, Keedy DA, Immormino RM, Kapral GJ, Murray LW, Richardson JS, Richardson DC. **2010**. MolProbity: all-atom structure validation for macromolecular crystallography. *Acta Crystallographica Section D: Biological Crystallography* **66**: 12–21.

Evans PR, Murshudov GN. **2013**. How good are my data and what is the resolution? *Acta Crystallographica. Section D, Biological Crystallography* **69**: 1204–1214.

Murshudov GN, Skubak P, Lebedev AA, Pannu NS, Steiner RA, Nicholls RA, Winn MD, Long F, Vagin AA. **2011**. REFMAC5 for the refinement of macromolecular crystal structures. *Acta Crystallographica Section D: Biological Crystallography* **67**: 355–367.
