# Supplementary material for: Arabidopsis downy mildew effector HaRxL106 suppresses plant immunity by binding to RADICAL‐INDUCED CELL DEATH1
Source: New Phytol. 2018 Aug 29;220(1):232–48. doi: 10.1111/nph.15277 (PMC6175486; doi:10.1111/nph.15277)
Supplement: Supplementary file 8 — Methods S1 Methods for Arabidopsis thaliana Paraquat treatment and SA quantification, nucleic acid extraction and quantification, protein extraction and purification, microscopy, protein crystallography and protein mass spectrometry. [file NPH-220-232-s008.pdf]

New Phytologist **Supporting Information Methods S1**

Article title: *Arabidopsis* Downy Mildew effector HaRxL106 suppresses plant immunity by binding to RADICAL-INDUCED CELL DEATH1

Authors: Lennart Wirthmueller, Shuta Asai, Ghanasyam Rallapalli, Jan Sklenar, Georgina Fabro, Dae Sung Kim, Ruth Lintermann, Pinja Jaspers, Michael Wrzaczek, Jaakko Kangasjärvi, Daniel MacLean, Frank L. H. Menke, Mark J. Banfield and Jonathan D. G. Jones

Article acceptance date: 09 May 2018

Methods S1. Methods for *Arabidopsis thaliana* Paraquat treatment and SA quantification, nucleic acid extraction and quantification, protein extraction and purification, microscopy, protein crystallography and protein mass spectrometry.

Paraquat treatment

Arabidopsis seedlings were sown on MS medium supplemented with 1 μM Paraquat. After 20 d the number of seedlings with expanded true leaves was counted.

Quantification of SA

Six-wk-old *Arabidopsis* plants were syringe-infiltrated with $1x10^8$ cfu ml^{-1} *Pst* DC3000 in 10 mM $MgCl_2$ or a 10 mM $MgCl_2$ mock solution. Samples were taken 24 h after infiltration including control samples from non-treated plants. Unconjugated SA was extracted and quantified as previously described (Aboul-Soud et al., 2004). Briefly, leaf tissue (0.2 g) was extracted in 1 ml 90% methanol following homogenization in liquid nitrogen. 3-hydroxybenzoic acid (HBA, Sigma) was used as an internal standard. The level of SA was determined by a

fluorescence detector (Shimadzu RF-20AXS, excitation at 305 nm and emission at 405 nm) in reverse-phase HPLC using a Nexera UHPLC (Shimadzu) system with a C18 (Kinetex 2.6µm XB-C18) column. We analyzed two to four technical replicates (independent samples from the same plant) as indicated in Fig. 2 in each of the three biological experiments (independent plant growth, treatment and sampling).

RNA extraction, cDNA synthesis and RNA-Seq transcriptome profiling

Five-wk-old *Arabidopsis* plants were syringe-infiltrated with $5x10^5$ cfu ml^{-1} *Pst* DC3000 at 12:00 (4 h after lights on). Rosette leaf samples from non-treated, mock- and bacteria-infiltrated plants were harvested 24 h later. Total RNA was extracted using the TRI reagent (Sigma) and 1-Bromo-3-chloropropane (Sigma), as per manufacturer's guidelines. RNA was precipitated with half volume of isopropanol and half volume of high salt precipitation buffer (0.8 M sodium citrate and 1.2 M sodium chloride). RNA samples were treated with DNaseI (Roche) according to the manufacturer's recommendation and phenol/chloroform extracted and ethanol precipitated. For qRT-PCR assays, mRNA was reverse transcribed using SuperScript II Reverse Transcriptase (Thermo Fisher). cDNA samples were diluted fivefold and 1 µl was used as template in a 20 µl qRT-PCR reaction. For RNA-seq, 3 µg of total RNA was used to generate first strand cDNA using a oligo(dT) primer comprising the P7 sequence of Illumina flow-cell. Double-stranded cDNA was synthesized as described previously (Okayama & Berg, 1982). Purified cDNA was subjected to Covaris shearing to a target size of 200 bp (parameters: Intensity – 5, Duty cycle – 20%, Cycles/Burst – 200, Duration – 90 s). End repairing and A-tailing of sheared cDNA was carried out as described by Illumina. Y-shaped adapters were ligated to A-tailed DNA and subjected to size selection on 1x TAE agarose gels. The gel-extracted library was PCR enriched and quantified using qPCR with previously sequenced similar size range Illumina library.

Transcriptome data has been deposited at NCBI's Gene Expression Omnibus (https://www.ncbi.nlm.nih.gov/geo/) with identifier GSE89402.

Yeast-two-Hybrid

All constructs for the Y2H assay were cloned into pDEST32 (bait) or pDEST22 (prey) vectors (Invitrogen) using Gateway® recombination. The RCD1, WWE-PARP (amino acids 1-471) and PARP-RST (amino acids 241-589) deletion constructs used for Fig. 6a have been published (Jaspers *et al.*, 2009). Two additional constructs, WWE (amino acids 1-155) and PARP (amino acids 247-472), were cloned into pDEST22 using the same strategy. For the Y2H assays in Fig. 9a-c the following RCD1 and SRO1 deletion constructs were generated: RCD1 WWE (amino acids 1-170), RCD1 WWE-linker (amino acids 1-265), RCD1 PARP (amino acids 265-460), RCD1 linker-PARP (amino acids 170-460), RCD1 WWE-PARP (amino acids 1-460), SRO1 WWE-linker (amino acids 1-262). For HaRxL106 constructs the pENTR plasmids carrying HaRxL106, HaRxL106ΔC or HaRxL106-Cterm58 were recombined into pDEST32. Yeast strain Mav203 was co-transformed with pDEST32 and pDEST22 plasmids and double-transformed yeast cells were selected on SD –Leu –Trp plates. Selected clones were grown in liquid SD –Leu –Trp medium at 30 °C for 48 h until cultures had reached saturation. The OD_{600} was adjusted to 0.1, and the yeast strains were plated in 10-fold serial dilutions onto SD –Leu –Trp –His medium containing 0, 1, 5, 10 or 20 mM 3-AT. Serial dilutions were also plated on SD –Leu –Trp medium to compare growth rate of the yeasts under non-selective conditions. The plates were photographed 3-5 d after plating.

qRT-PCR

qRT-PCR was performed using 10 µl SYBR Green qPCR premix (Sigma) and the following oligonucleotides: *EF1α*-fw CAGGCTGATTGTGCTGTTCTTA, *EF1α*-rv GTTGTATCCGACCTTCTTCAGG, *PR1*-fw ATGAATTTTACTGGCTATTCTC, *PR1*-rv AGGGAAGAACAAGAGCAACTA. qRT-PCR reactions were run in duplicates or in triplicates on a CFX96 Touch™ Real-Time PCR Detection System (Bio-Rad) using the following program: (1) 95 °C, 3 min; (2) [95 °C for 30 s, 60 °C for 30 s, 72 °C for 30 s] ×41, (3) 72 °C for 10 min followed by (4) a melting curve analysis from 55 °C to 95 °C. The data were analyzed using CFX Manager™ software (Bio-Rad). For each cDNA sample, we performed two or three technical replicates (independent PCR reactions from the same cDNA). The average relative transcript levels from three independently sown, treated and sampled biological replicates were analyzed using one-way ANOVA followed by a Tukey-Kramer post hoc test.

Transient expression

RCD1:GFP and SRO1:GFP constructs were generated by cloning their coding sequences into pENTR/D-TOPO and recombining these plasmids with pK7FWG2 (Karimi *et al.*, 2002). *A. tumefaciens* GV3101::pMP90 and GV3101::pMP90RK strains were grown on selective plates, resuspended in 10 mM $MgCl_2$ 10 mM MES pH 5.6 and incubated with 100 µM acetosyringone for 2 h at RT. Prior to infiltration, each strain was mixed with *A. tumefaciens* strain GV3101::pMP90 expressing the silencing suppressor 19K at a ratio of 1:2[19K]. For co-expression, strains were mixed in a 1:1:2[19K] ratio. We infiltrated leaves of 4-wk-old *N. benthamiana* plants with a needleless syringe and harvested the leaves 48–72 h later.

Protein extraction from *A. thaliana*, *N. benthamiana*, immunoprecipitation and western blot

Protein extracts were prepared by grinding *A. thaliana* or *N. benthamiana* leaf material in liquid nitrogen to a fine powder followed by resuspension in extraction buffer [50 mM Tris, 150 mM NaCl, 10% glycerol, 1 mM EDTA, 5 mM DTT, 1× protease inhibitor cocktail (Sigma #P9599), 0.2% NP-40, pH 7.5] at a ratio of 2 ml buffer per 1 g leaf material. For experiments that included RCD1 (domains) the proteasome inhibitor MG132 (10 μM) and phosphatase inhibitors NaF (10 mM) and Na_3VO_4 (1 mM) were added to all buffers. Crude protein extracts were centrifuged at 20.000 x ***g*** 4°C 20 min and the supernatant was either boiled in sodium dodecyl sulphate (SDS) sample buffer for western blots or used for immunoprecipitation. For western blots proteins were separated by SDS-PAGE and electro-blotted onto polyvinylidene difluoride membrane (Millipore). Antibodies α-HA 3F10 (Roche), α-GFP 210-PS-1GFP (Amsbio), α-RFP-biotin ab34771 (Abcam) were used for detection. For immunoprecipitation a fraction of the supernatant was saved as 'input' sample and 15 μl GFP-beads (GFP-Trap_A; Chromotek) were added to the remaining supernatant. The volume for immunoprecipitation from *N. benthamiana* for western blots was 1.4 ml. The volume for immunoprecipitation from *A. thaliana* was 4 ml. The volume for immunoprecipitation experiments coupled to mass spectrometry was 15-25 ml. Following incubation of the samples on a rotating wheel at 4°C for 2 h the beads were collected by centrifugation at 1200 x ***g*** and 4°C for 1.5 min. The beads were washed 3 times with 1 ml extraction buffer and then boiled in SDS sample buffer to elute proteins from the beads.

Thermal shift assays

Thermal shift assays were performed in a CFX96 Touch™ Real-Time PCR Detection System (Bio-Rad) as described by (Vivoli *et al.*, 2014) with minor modifications. Briefly, 23 μl reactions containing the PARP domains of HsPARP1 [L713F mutant, (Langelier *et al.*, 2012)] or RCD1 at

a final concentration of 0.11 mg ml^{-1} were prepared in reaction buffer [40 mM HEPES pH 7.5, 150 mM NaCl, 6.6x SYPRO Orange (Thermo Fisher)]. 6(5H)-phenanthridinone (Sigma) was added from a 25 mM stock solution in DMSO to final concentrations of 2 nM to 2 mM or DMSO was used as a control. The total reaction volume was 25 µl. Unfolding of the proteins was determined by running a thermal denaturation program with 0.5 °C temperature increase per cycle followed by measuring SYPRO orange fluorescence in FRET mode of the instrument. For each protein/ligand combination, we measured three independent technical replicates and plotted the first derivative of the fluorescence mean values over temperature using CFX Manager™ software (Bio-Rad). Table S12 shows data from three independent biological replicates.

Confocal microscopy

Arabidopsis leaf discs were mounted onto microscopy slides in 50% glycerol or water and analyzed on a Leica DM6000B/TCS SP5 confocal microscope with the 488 nm as excitation wavelengths for GFP.

Recombinant expression and purification of PARP domains from *E. coli*

The expression construct for the human PARP1 PARP domain (amino acids 662-1014; L713F mutant) has been described (Langelier *et al.*, 2012). The PARP domain of RCD1 (amino acids 269-460) was cloned into the pOPINF vector (Berrow *et al.*, 2007) linearized by KpnI/HindIII digest using Gibson assembly. The expression construct was transformed into SoluBL21 DE3 *E. coli* cells (Genlantis, NEB). For protein expression four 1 l cultures were grown in LB medium at a temperature of 37°C to an OD_{600} of 1.0 – 1.2. The cultures were cooled to 18°C before expression was induced by the addition of 0.5 mM IPTG for 16 h. Cells were pelleted by centrifugation (5000 x ***g***, 4°C , 12 min) and the pellets were resuspended in buffer A (50 mM

Tris-HCl, 0.3 M NaCl, 20 mM imidazole, 5% glycerol, 50 mM glycine, pH 8.0) supplemented with 0.1% Polyethylenimine and 1x cOmplete™ EDTA-free protease inhibitor cocktail (Roche). Cells lysis was induced by addition of lysozyme (1 mg ml^{-1} final concentration, RT, 15 min) followed by sonication. Insoluble proteins and cell debris were removed by centrifugation (30.000 x ***g***, 4°C , 20 min) and the supernatant was loaded onto a 5 ml HisTrap HP IMAC column (GE Healthcare). The column was washed with buffer A until the A_{280} reached 25 mAU and proteins were eluted using buffer B (50 mM Tris-HCl, 0.3 M NaCl, 20 mM imidazole, 5% glycerol, 50 mM glycine, 500 mM Imidazole, pH 8.0). The elution from the IMAC column was injected onto a size exclusion chromatography column [Superdex 75 26/60 PG column (GE Healthcare) pre-equilibrated in 20 mM HEPES, 150 mM NaCl, pH 7.5]. PARP domains eluting from the column were concentrated to 0.5-1 mg ml^{-1} (HsPARP1) and used for thermal shift assays. For crystallization of the RCD1 PARP domain, the His6-tag was cleaved using 3C protease. The protein was run through a 5 ml HisTrap HP IMAC column in buffer A to remove the His6 tag and residual uncleaved fusion protein, followed by injection onto the Superdex 75 26/60 PG column and eluted as above. The protein was concentrated to 35 mg ml^{-1} using Vivaspin 20 and 2 columns (Sartorius) with a molecular weight cut-off of 5 kDa and flash-frozen in liquid nitrogen. Seleno-Met-labelled RCD1 PARP protein was produced using feedback-inhibition and purified as described above for native RCD1 PARP.

Crystallization and data collection

The RCD1 PARP domain was used for crystal screens at a concentration of 35 mg ml^{-1}. Crystals of native and Seleno-Met-labelled protein grew in 0.1 M HEPES pH7.5, 1.6 M Ammoniumsulfate, 2% PEG1000 in hanging drops at 20°C. The crystals were harvested in Paratone-N oil (Hampton) and frozen in liquid nitrogen. Data collection was performed on

beamlines i04 (native) and i24 (Seleno-Met) at Diamond Light Source, Oxford, UK. X-ray data were processed with iMosflm (Battye *et al.*, 2011) and scaled with Aimless (Evans & Murshudov, 2013) from the CCP4 suite (Collaborative Computational Project, Number 4, 1994). For X-ray data collection statistics see Table S11. The RCD1 PARP structure was solved by single wavelength anomalous dispersion using Phaser (McCoy, 2007). Iterative building and refinement cycles with Coot (Emsley *et al.*, 2010), Refmac5 (Murshudov *et al.*, 2011) and Phenix (Adams *et al.*, 2010) were used to obtain the final model with statistics given in Table S11. Validation tools in Molprobity (Chen *et al.*, 2010) and Coot were used to analyze the final structure. 3D visualizations of protein structures were prepared using PyMOL software v1.7.2 (http://sourceforge.net/projects/pymol/). Reflection data and the RCD1 PARP domain structure have been deposited at the Protein Data Bank with identifier 5NGO.

Protein mass spectrometry

Samples for LC-MS analysis were prepared by excising bands from one dimensional SDS-PAGE gels stained with colloid Coomassie Brilliant Blue (Instant Blue, Expedeon). The gel slices were destained with 50% Acetonitrile and cysteine residues modified by 30 min reduction in 10 mM DTT followed by 20 min alkylation with 50 mM chloroacetamide. After extensive washing with 30% Acetonitrile and dehydration with 100% acetonitrile the pieces were incubated with 100 ng of trypsin (Promega) in 100 mM ammonium bicarbonate, 10% acetonitrile at 37ºC overnight.

LC-MS/MS analysis was performed using a hybrid mass spectrometer Orbitrap Fusion and a nanoflow UHPLC system U3000 (Thermo Scientific). The generated peptides were applied to a reverse phase trap column (Acclaim Pepmap 100, 5 µm, 100 µm x 20 mm) connected to an analytical column (Acclaim Pepmap 100, 3 µm, 75 µm x 500 mm; Thermo Scientific). Peptides were eluted in a gradient of 9-50% acetonitrile in 0.1% formic acid (solvent B) over 50 min

followed by a gradient of 50-60% B over 3 min at a flow rate of 300 nL min^{-1}. The mass spectrometer was operated in positive ion mode with nano-electrospray ion source with an ID 0.01 mm fussed silica PicoTip emitter (New Objective). Voltage +2.2 kV was applied via conductive T-shaped coupling union. Transfer capillary temperature was set to 320ºC, no sheath gas, and the focusing voltages were in factory default setting. Method MS events consisted from full scan in Orbitrap analyzer followed by two collisions of 'softer' CID and more 'energetic' HCD to maximize the chances to acquire spectra with structurally important information. Orbitrap full scan resolution of 120000, mass range 300 to 1800 m/z automatic gain control (AGC) target 200000 and maximal infusion time 50 ms were set. Data-dependent algorithm MS/MS fragmentation of large number of precursor ions was used. The dynamic exclusion 30 s, 'Top speed' precursor selection method within 3 s cycle between full scans and 'Universal method' for infusion time and AGC calculation were used. The isolation width 1.6 m/z and normalized collision energy of 30% were set for both CID and HCD collisions. Only the precursor ions with positive charge states 2 – 7 and intensity greater than 10000 were selected for MS/MS fragmentation.

Software processing and peptide identification

Peak lists in the form of Mascot generic files (mgf files) were prepared from raw data using MS Convert (Proteowizard project) and sent to peptide match search on the Mascot server using Mascot Daemon (Matrix Science). Peak lists were searched against *N. benthamiana* or *A. thaliana* databases. Tryptic peptides with up to 2 possible miscleavages and charge states +2, +3, +4 were allowed in the search. The following modifications were included in the search: oxidized methionine (variable), carbamidomethylated cysteine (static). Data were searched with a monoisotopic precursor and fragment ion mass tolerance 10 ppm and 0.6 Da respectively. Mascot

results were combined in Scaffold (Proteome Software) and exported to Excel (Microsoft Office) for sample-to-sample comparison. In Scaffold, the peptide and protein identifications were accepted if probability of sequence match and protein inference exceeded 95% and 99%, respectively. At least 2 identified peptides per protein were required. Protein probabilities were calculated by the Protein Prophet algorithm; proteins that contained similar peptides and could not be differentiated based on MS/MS analysis alone were grouped to satisfy the principles of parsimony (Searle, 2010).

SI References

Aboul-Soud M, Loake GJ. 2004. Measurement of Salicylic Acid by a High-Performance Liquid Chromatography procedure based on ion-exchange. *Chromatographia* **59**: 129-133.

Adams PD, Afonine PV, Bunkóczi G, Chen VB, Davis IW, Echols N, Headd JJ, Hung L-W, Kapral GJ, Grosse-Kunstleve RW, *et al.* 2010. PHENIX: a comprehensive Python-based system for macromolecular structure solution. *Acta Crystallographica. Section D, Biological Crystallography* **66**: 213–221.

Battye TGG, Kontogiannis L, Johnson O, Powell HR, Leslie AGW. **2011**. iMOSFLM: a new graphical interface for diffraction-image processing with MOSFLM. *Acta Crystallographica Section D: Biological Crystallography* **67**: 271–281.

Berrow NS, Alderton D, Sainsbury S, Nettleship J, Assenberg R, Rahman N, Stuart DI, Owens RJ. **2007**. A versatile ligation-independent cloning method suitable for high-throughput expression screening applications. *Nucleic Acids Research* **35**: e45.

Chen VB, Arendall WB, Headd JJ, Keedy DA, Immormino RM, Kapral GJ, Murray LW, Richardson JS, Richardson DC. **2010**. MolProbity: all-atom structure validation for macromolecular crystallography. *Acta Crystallographica Section D: Biological Crystallography* **66**: 12–21.

Emsley P, Lohkamp B, Scott WG, Cowtan K. **2010**. Features and development of Coot. *Acta Crystallographica. Section D, Biological Crystallography* **66**: 486–501.

Evans PR, Murshudov GN. **2013**. How good are my data and what is the resolution? *Acta Crystallographica. Section D, Biological Crystallography* **69**: 1204–1214.

Jaspers P, Blomster T, Brosché M, Salojärvi J, Ahlfors R, Vainonen JP, Reddy RA, Immink R, Angenent G, Turck F, *et al.* 2009. Unequally redundant *RCD1* and *SRO1* mediate

stress and developmental responses and interact with transcription factors. *The Plant Journal: For Cell and Molecular Biology* **60**: 268–279.

Karimi M, Inzé D, Depicker A. **2002**. GATEWAY vectors for *Agrobacterium*-mediated plant transformation. *Trends in Plant Science* **7**: 193–195.

Langelier M-F, Planck JL, Roy S, Pascal JM. **2012**. Structural basis for DNA damage-dependent poly(ADP-ribosyl)ation by human PARP-1. *Science (New York, N.Y.)* **336**: 728–732.

McCoy AJ. **2007**. Solving structures of protein complexes by molecular replacement with Phaser. *Acta Crystallographica. Section D, Biological Crystallography* **63**: 32–41.

Murshudov GN, Skubak P, Lebedev AA, Pannu NS, Steiner RA, Nicholls RA, Winn MD, Long F, Vagin AA. **2011**. REFMAC5 for the refinement of macromolecular crystal structures. *Acta Crystallographica Section D: Biological Crystallography* **67**: 355–367.

Okayama H, Berg P. **1982**. High-efficiency cloning of full-length cDNA. *Molecular and Cellular Biology* **2**: 161–170.

Searle BC. **2010**. Scaffold: a bioinformatic tool for validating MS/MS-based proteomic studies. *Proteomics* **10**: 1265–1269.

Vivoli M, Novak HR, Littlechild JA, Harmer NJ. **2014**. Determination of protein-ligand interactions using differential scanning fluorimetry. *Journal of Visualized Experiments: JoVE*: 51809.
